# Himalayan ecosystem services and climate change driven agricultural frontiers: a scoping review

**DOI:** 10.1007/s43621-022-00103-9

**Published:** 2022-10-18

**Authors:** Krishna Bahadur KC, Edan Tzadok, Laxmi Pant

**Affiliations:** 1 Department of Geography, Environment and Geomatics, University of Guelph, Guelph, Ontario N1G 2W1 Canada; 2 Natural Resources Institute, University of Greenwich, Central Avenue, Chatham Maritime, ME4 4TB UK

## Abstract

Climate change, rising temperatures, snow melts and more frequent droughts and floods are disproportionately affecting food and water security, habitat health, and agricultural productivity in the Himalayan region. These climatic changes are negatively impacting productivity of staple crops including wheat, maize, and rice at lower altitudes, but may provide opportunities to utilize Climate Change Driven Agricultural Frontiers [CCDAFs] at higher altitudes. Agricultural expansion into CCDAFs paired with behavioural shifts such as replacing traditional crop systems with commercial crops will predominantly affect forests, water resources, and soil health, which are already negatively affected by climate change unless adaptation options are directed to just and sustainable agroecological transitions. By trading regulating, supporting, and cultural services for food and water provisioning services, as are evident in land sparing strategy, the utilization of CCDAFs will have long-term implications for the sustainability of mountain farming systems. Climate change is affecting Himalayan agriculture, food security, and ecosystem services, and scientific literature predominantly focus on one of these topics in isolation, occasionally connecting results to another topic. By classifying literature as predominantly agriculture, food security, or ecosystem service themed, this scoping review identifies sources with multiple dominant themes and explores how the relationships between these topics are represented in literature to provide research based evidence to promote the future expansion of agriculture that is low-carbon, just and sustainable. Gaps in the literature reveal that research is needed on the extent of CCDAFs in the Himalayas and the potential trade-offs on utilizing the frontier areas.

**Supplementary Information:**

The online version contains supplementary material available at 10.1007/s43621-022-00103-9.

## Introduction

The Himalayan range is an extensive mountain ecosystem highly vulnerable to climate change [1]. This region is warming faster than the rest of the world, and the Trans-Himalaya, which lies on the southern edge of the Tibetan Plateau, in particular is warming at a faster rate than the rest of the Himalayas [2]. Himalayan warming has caused glacial retreat, glacial lake expansion and outburst, rapid snow melt, more frequent extreme events including precipitation, drought and desertification, a transition from a snow to rain dominant precipitation system, forward shift in precipitation, earlier flowering and fruiting, and upslope treeline shift [1–4]. Consequently, many lowland species are expanding their ranges to higher elevations to take advantage of warming temperatures and longer frost-free days [1, 3, 5]. As well, the extent of grasslands and forests are receding due to climate change, which has implications for biodiversity conservation [1]. These changes impact important ecosystem services that support Himalayan communities.

We define ecosystem services [ES] according to the Common International Classification of Ecosystem Services [CICES V5.1], which is a system that outlines and maps ES classifications in detail [6]. The CICES V5.1 defines ecosystem services as “the contributions that ecosystems make to human well-being” [6]. This review primarily focuses on ecosystem services that fall under *provisioning* and *regulation and maintenance* sections of the CICES classification system because these are most relevant to agriculture and food security.

Changing environmental conditions are causing food production challenges related to water scarcity, timing of harvest, increased pests and diseases, altered phenology, and lack of feed and fodder [7, 8]. However, climate change may allow farmers to grow crops in areas that were not previously used for agriculture. As the climate warms, new land is projected to become suitable for agriculture, providing opportunities for agricultural expansion globally on Climate Change Driven Agricultural Frontiers (CCDAF)—described as areas that will become suitable for agriculture in the future due to changing climatic conditions [9]. According to Hannah et al. [9], 10.3–24.1 million km^2^ of CCDAFs could arise globally by 2060–2080. Agricultural expansion into CCDAFs could help address food insecurity by creating additional opportunities for farmers; however, utilizing these areas for large scale commercial agriculture carries consequences for the balance of regional ecosystem services [9].

As climate change becomes an increasingly prevalent threat to agriculture, more and more communities are facing food insecurity and meeting food needs of a rapidly growing population is becoming increasingly difficult [8]. In Upper-Mustang, one of the arid Trans-Himalayan regions in Nepal, for example, approximately 50% of households experience some level of food insecurity because of high market prices of food transported from lower altitudes and low-quality farmland [10]. According to FAO [11], globally, 51% of people facing chronic hunger reside in the Hindu-Kush Himalayas from Afghanistan to northern Pakistan and Tajikistan. As well, about 1.3 billion people in South Asia and the Himalayan region depend on ecosystem services from the Himalayas [12]. Forests and water resources are particularly important as they provide regulatory and provisioning ecosystem services such as soil health and carbon sequestration and agriculture which contribute to Himalayan livelihoods [13]. For instance, Himalayan communities largely rely on agriculture as a main source of livelihood, and farmers face the complex challenge of meeting food production needs while facing the impacts of climate change, water stress, and exhaustion of agricultural land [1, 8, 14].

Climate change is affecting various aspects of environmental systems and human livelihoods in the Himalayas including agriculture, food security, and ecosystem services. Scientific literature rarely examines the interactions between these themes; rather, the literature predominantly focuses on one of these topics in isolation, occasionally connecting results to another topic. This scoping review uses methods that classify literature as predominantly agriculture, food security, or ecosystem service themed to identify sources with multiple dominant themes and to explore how the relationships between these topics are represented in the literature. This review also identifies research gaps accordingly and provides suggestions for future research on CCDAFs in the Himalayas. Given the importance of emerging climate change driven agricultural frontiers, connections between ecosystem services, agriculture, and food security must be understood in depth as they will help determine the impacts of utilizing agricultural frontiers. This review aims to answer the following questions:

1. What are the impacts of climate change on food security and agriculture in the Himalayas?
2. What is the future scope of promoting low-carbon agricultural expansion in the climate change driven Himalayan agricultural frontiers?
3. Howe does ecosystem services support agri-food system sustainability in the climate change driven Himalayan agricultural frontiers?

## Methods

**Fig. 1:**
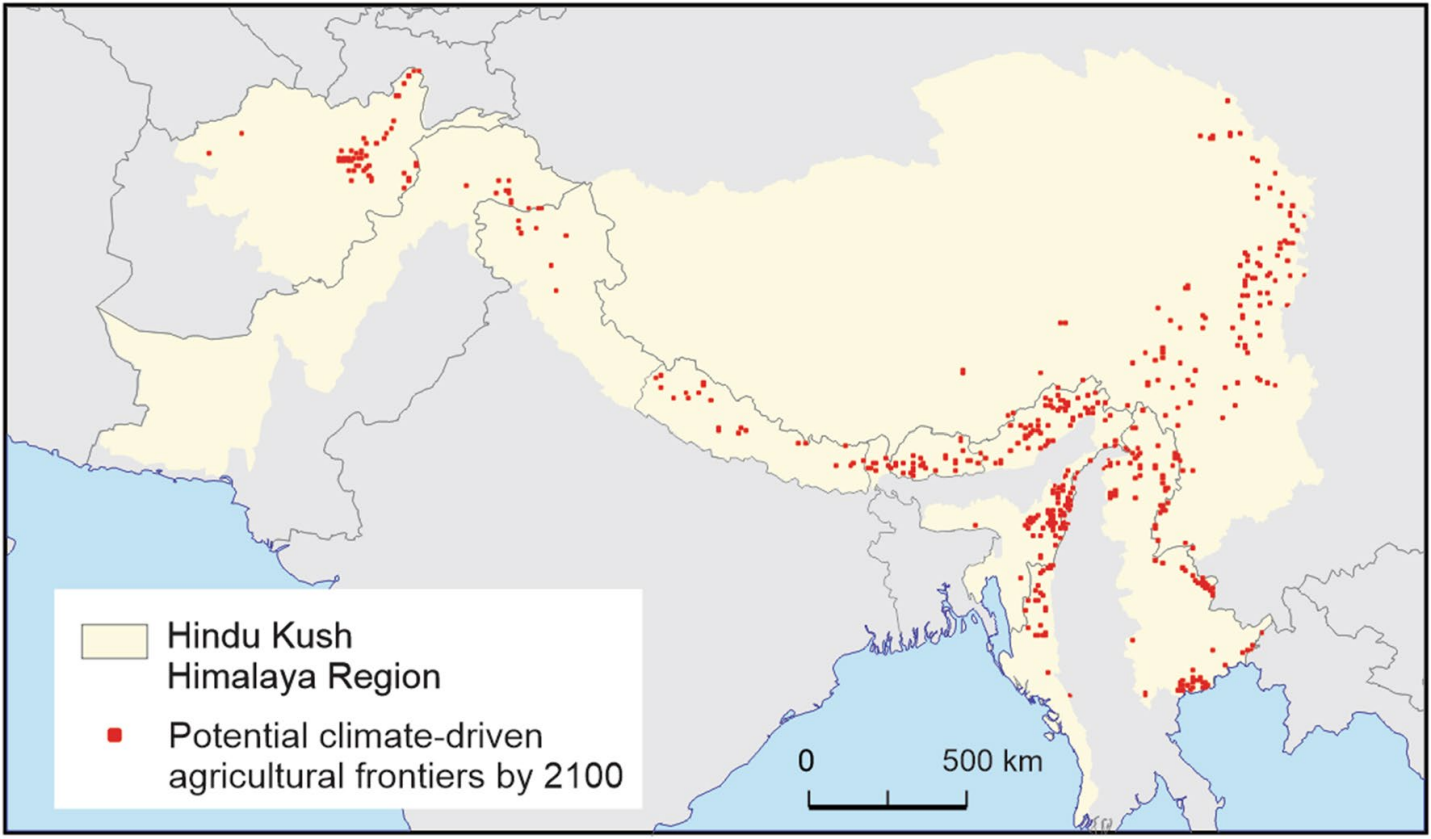
Hindu Kush Himalaya region showing the potential climate driven frontier by 2100 (Adapted from Hannah et al. [9], KC et al. [19], KC et al. [20])

First, we chose Hindu Kush Himalaya region (Fig. 1) as the study area for this review. Our review follows scoping methods outlined by Arksey & O’Malley [15–17], and is based on the methods used by Green et al. [18]. We followed the PRISMA-ScR (Preferred Reporting Items for Systematic reviews and Meta-Analyses extension for Scoping Reviews) checklist using the following steps: (1) identify research questions; (2) conduct keyword searches in electronic databases using exclusion criteria and review article reference lists; (3) perform full-text reviews of literature; (4) thematic grouping of sources; and (5) report results. Details of each step of the review process are described below.

1. *Identification of research questions:* Guiding research questions were determined based on knowledge from previous research by Hannah et al. [9] and KC et al. [19] on Climate Change Driven Agricultural Frontiers, with the intent of determining gaps in knowledge and implications for future research in these areas.
2. *Identification and screening of literature:* First, both Web of Science [WoS] and PubMed was used to identify peer-reviewed literature. Second, Google Scholar was used to identify significant grey literature, such as Government reports and reputable non-government reports [e. g., ICIMOD], and peer-reviewed literature that was not found in the WoS and PubMed search. Combinations of the following words were used for the keywords search: climate change, Himalaya, Trans-Himalaya, agriculture, agriculture productivity, crop suitability, crop yield, food security, ecosystem service (see Table 3). Keyword combinations were searched in both WoS and PubMed under “title/abstract/keywords”. Results were first sorted in WoS by relevance and screened to include only the first 20 listed sources. The same was done for the results in PubMed.This was done because only the most relevant results to the keywords searched were needed Then, results were screened by publication year,.Sources from 2001 to 2022 were reviewed; however sources published between 2001 and 2010 were chosen only if they were cited more than 5 times. This was done to ensure that older sources being reviewed were deemed significant by the scientific community. Papers were then chosen for full-text review if the title and abstract indicated that the paper addressed relationships between climate change, agriculture, food security, crop productivity, or ecosystem services, and if results could be applied in the context of agricultural expansion frontiers. The reference lists of the resulting records were examined for additional relevant citations based on relevance of title and year.
3. *Full-text reviews:* The collected literature (n = 53) was reviewed and the following information was recorded from each source: author, year, location, aims, methods, and important results.
4. *Thematic grouping of sources:* The literature was grouped into three collections by the dominant theme of each paper: (i) agriculture, (ii) food security, or (iii) ecosystems services. Sources were also grouped by type of methods used (i) GIS, (ii) interviews, or (iii) other. If a paper had multiple dominant themes (i.e., discussed agriculture and food security equally), it was listed under both themes. The same was true for papers with multiple methods.
5. *Report results:* A written summary of the literature is presented in the following section and results are shown in tables below.

## Results

### Quantitative results of thematic grouping and keyword search

A total of n = 53 sources were identified and reviewed. Dominant themes are shown in Table 1 along with the number and percent of records that contain each dominant theme. The thematic grouping process revealed that majority of the reviewed sources (n = 31 or 48%) predominantly addressed ecosystem functions, followed by agriculture (n = 16 or 40.5%), and food security (n = 9 or 14%). There were seven (n = 11) sources that predominantly addressed more than one theme.

**Table 1:** Count and percent of sources predominantly discussing each main theme

Dominant theme	Count	Percent (%)
Agriculture	25	38
Food security	9	14
Ecosystem function	31	48

Since several sources contained more than one dominant theme, the total count in Table 2 is larger than the total number of sources (n = 53) and the sum of the percentages is greater than 100. Table 2 shows the number and percent of sources that were categorized based on methods used—GIS, interview, or other. The “other” category encompasses government and NGO reports as well as studies that analyzed field data or climate data without using GIS. Sources that used multiple methods were listed under all relevant categories, therefore the summed count from the table is over the total number of sources (n = 53) and the percentages are greater than 100. A total of n = 16 (30%) sources used methods that included interviewing individuals, n = 8 (15%) sources used GIS analysis methods, and n = 30 (55%) sources were categorized as Other. There was n = 2 sources that used a combination of GIS and interview methods. Comparatively, GIS analysis was the least used method.

**Table 2:** Count and percent of sources that collected data using GIS analysis, interviews, or other methods

Method	Count	Percent (%)
GIS analysis	8	15
Local interviews	16	30
Other	30	55

Table 3 shows the quantity of results from Web of Science PubMed keyword searches. Of all the keyword combinations, “climate change and Himalaya* and ecosystem service*” produced the greatest number of results (n = 131, Web of Science n = 131. PubMed n = 12) and “climate change and Himalaya* and crop suitability” produced the least results (n = 10 Web of Science n = 8. PubMed n = 2). “Agriculture and Trans-Himalaya*” produced the second least amount of results (n = 47, Web of Science n = 21. PubMed n = 26), indicating that the body of research focused on agriculture in the Trans-Himalaya is not as well-developed or extensive as the research in the general Himalaya area. Overall, the quantity of sources found indicates that there is a relatively well-developed body of literature on ecosystem services related to climate change in the Himalayas compared to studies on crop suitability in the Himalayas. It is also important to note that 11 sources were published between 2019 and 2021. This indicates that there is new emerging research on agriculture, food security, ecosystem services, and climate change in the Himalayas.

**Table 3:** Combinations of keyword search terms and number of results from Web of Science and PubMed

Keyword 1	Keyword 2	Keyword 3	Number (Web of Science)	Number (PubMed)
Climate change	Himalaya*	Agriculture productivity	44	31
Climate change	Himalaya*	Crop yield	70	9
Climate change	Himalaya*	Crop suitability	8	2
Climate change	Himalaya*	Ecosystem service*	131	12
Agriculture	Himalaya*	Ecosystem service*	53	19
Agriculture	Trans Himalaya*		21	26

### Qualitative results and connections in literature

Sources that contained more than one dominant theme presented links between the multiple themes. Sources with only one dominant theme also made connections to other themes in a secondary sense. These connections are outlined below as they relate to the three driving research questions of this study, and are presented in Table 4.

**Table 4:** Explanation of interactions between ecosystem services, food security, crop productivity, and agriculture across research questions 1–3

Ecosystem service (ES)	Description	Q1: Impact on food security	Q2: Impact on crop productivity and suitability	Q3: Agriculture to ecosystems
Forest resources	Understory and canopy plants	Edible and medicinal plants, increased local income	Soil stability, agroforestry, water retention, feed for livestock that produce manure	Loss and fragmentation
Biodiversity	Species, communities, or genetic biodiversity	Forest biodiversity supports local food security Crop genetic diversity increases agricultural resilience	Diverse crop systems are more productive	Biodiversity loss from agriculture expansion Agroforestry and crop-diversification contribute to biodiversity
Water resources	Precipitation, glacier retreat, glacial lake outburst floods, droughts, hydrological cycle disruption	Water scarcity, floods, droughts negatively impacts food security	Precipitation variability, floods, and droughts negatively impact productivity	Increased run-off from some cash crops Decreased run-off from agroforestry systems
Soil health	Soil structure, erosion control, fertility	Stability of crop yield over time	Decreased productivity from erosion and landslides	Soil loss and erosion from some cash crops Increased soil stability and fertility from agroforestry systems
Climate regulation	GHG emissions, CO_2_ sequestration, regulation of climate variability, e.g., reduction in temperature, flood regulation, wind breaks	Agroecosystem resilience Stability of food supply	Crop yield stability Reduced crop damage by biotic and abiotic stressors	Improved supply of regulating ecosystem services

#### Q1: Impact of climate change on food security and agriculture in the Himalayas

Sources that discuss food security in mountain regions explain that high mountain communities, including the Trans-Himalaya, are significantly more vulnerable to food insecurity compared to lowland areas because they face low productivity, climate and terrain limitations, natural hazards, poor infrastructure, difficulty accessing markets, and high production and transportation costs, and experience higher rates of micronutrient deficiency [15, 21]. Multiple studies (n = 5) noted a decrease in area under traditional crops in the Himalayas and Trans-Himalaya as farmers adopted modern crop varieties in attempts in increase income from sales in the market [5, 22, 23]. Traditional and staple crops replaced by modern crop varieties include horse gram, buckwheat, millet, soybean, sweet potato, barley, amaranths, chenopods, black pea, and potato. Many of these traditional crops are adapted to arid climate and grows on marginal lands with poor soil fertility. One study noted a connection between this transition to agricultural modernisation and the loss of ecosystem services. Additionally, nearly a third of the literature (n = 16 or 30%) used data collected from interviews to determine how local farmers in mountain communities are perceiving and adapting to climate change, including the seemingly maladaptive practice of modern crop variety adoption without discussing agroecological transition potential, such as agroforestry and perennial cropping.

#### Q2. Impact of climate change on future crop suitability and opportunities for agricultural expansion in the Himalayas

Crop suitability projections are mentioned by several sources (n = 8) which were grouped under the agriculture or food security themes. The general consensus from the literature is that crop suitability and yields are expected to increase at higher altitudes and new land may become available for agriculture because of warming temperatures [8, 15, 24–28]. Of all the sources reviewed, only 1 study did a crop suitability analysis under different representative concentration pathways RCP scenarios using GIS [29]. One other study used GIS to conduct a crop yield analysis and connected results to crop nutrition [25]. Studies also used interview methods to gain an understanding of how climate change impacts crop yield and productivity. Results show trends of farmers noticing a decrease in crop productivity and farm income as a result of climate change and water stress.

#### Q3. Connections between ecosystem services and agriculture in the Himalayas

Agriculture and ecosystem services are intricately connected in the Himalayas. There was n = 6 sources that fell under both agriculture and ecosystem services themes. Forest resources are highly discussed in the literature and studies shows that forests provide both provisioning and regulatory services through soil stability, efficient nutrient cycling, and decreased run-off, which contributes to agricultural productivity and food security. Biodiversity in both agroecosystems and natural ecosystems is important for the maintenance of these systems and human livelihoods. Several sources present agricultural expansion, agricultural intensification, and climate change as drivers of forests degradation and biodiversity loss [15, 30–32]. Agricultural systems may also contribute positively to forest resources and biodiversity through agroforestry and crop-diversification to grow perennial crops. Water resources are presented in the literature as being an important ecosystem service that is negatively affected by climate change, in turn having consequences for agriculture. Crop productivity will be negatively affected by more intense and frequent droughts and floods caused by climate change. Agricultural systems may increase run-off if intensive cultivation of modern crop varieties are involved, or they may decrease run-off if agroforestry methods or other perennial cropping systems are practiced. Sources that link agriculture and ecosystem services also mentioned that soil health is impacted by intensive agriculture as it impacts soil stability, soil fertility and biophysical properties. On the other hand, the literature emphasizes that agroforestry methods have a positive impact on soil stability and fertility. Lastly, agriculture impacts climate regulation services as croplands have higher rates of greenhouse gas emissions.

## Discussion

### Climate change, agriculture, and food security in the Himalayas

#### Impact of climate change on crop production

Climate change is impacting microclimates in the Himalayas, in turn affecting crop suitability, productivity, and yield. A 30% decrease in crop yield can be expected by the mid-twenty-first century in Central and South Asia, leading to lower income and lower food availability [4], and ultimately contributing to food insecurity. Farmers throughout the Hindu-Kush Himalaya have reportedly noticed a decrease in crop production and farm income as a result of climate change [21]. A crop yield analysis in Nepal showed that yield of maize, rice, and wheat would decrease by 2100 because of climate change, and that these crops would benefit from being grown in higher altitudes as it would increase yield and nutritional value [25]. Another study did a crop suitability analysis in Nepal and found that suitability for 5 major crops (wheat, potato, finger millet, maize, and rice) will decrease at lower elevations and increase at higher elevations because of climate change [29].

In addition, several studies recorded that farmers have begun cropping at higher altitudes as microclimates become suitable for agriculture. Rasul [8] found that fruit farming has increased in India, Pakistan, Bhutan, and Nepal—indicating that they are taking advantage of suitable climatic conditions at higher altitudes. Apples in Himachal Pradesh, India are now being grown at over 3500 m elevation since 2014 because of warming temperatures [23]. A land use study by Quasim et al. [32] also recorded agricultural expansion in higher altitudes in Pakistan. According to Poudel’s [33] study of the Nhāson village in the Trans-Himalaya region of Nepal, people are now growing apple, maize, and green vegetables in areas where they were not grown previously. Farming at higher altitudes may become more common in the future as climate change progresses; thus, it is important that research evidence is generated to inform policy that enable the right type of agricultural expansion –- is promoted in the climate change driven agricultural frontiers in the high altitudes.

Although multiple sources in this review mention that new land may become suitable for agriculture at higher altitudes as a result of climate change, a very small portion of the literature quantified future crop suitability in the Himalayas. No studies quantified the extent of climate change driven agricultural frontiers. Specific information on crop suitability and agricultural frontiers is needed, especially for the Trans-Himalaya region as this is where new agricultural frontiers will most likely develop and have greater economic importance and potential for carbon emission. This information would be useful in determining the extent and locations of potential CCDAFs in the region and their environmental and economic impacts.

#### Food security and modern varieties of cash crops

With the growing concerns of food availability and income, farmers have begun cultivating more modern varieties of cash crops in an attempt to maximize farm income. Sharma et al. [5] noted that the area under traditional crops in the Sikkim Himalaya, including horse gram, buckwheat, finger millet, soybean, sweet potato, barley, amaranths, and chenopods, has decreased by 50–60% since late 1970s. The same study found that the area under staple crops including maize, rice, and pulses is also declining [5]. Likewise, buckwheat, hog millet, foxtail millet, and various other traditional crops are being replaced by potato which is valued in the market [22]. Traditional crops in the Trans-Himalaya such as barely, local potato, and black pea are being replaced by modern cash crops [23]. While the adoption of commercial crops may provide some financial benefit in the short term, it can pose environmental risks [22]. First, the traditional crops mentioned above have high nutritional and medicinal value as well as high market value, and they are even considered neutralizers of negative economic impacts of globalization and environmental change because minor millet and beans are drought tolerant and adapted to less fertile soils [5]. Thus, a reduction in traditional crops has negative implications for financial security and food security. Second, increased soil loss and run-off are associated with intensive cultivation of modern crop varieties, including commercial potato farming [22]. It is important that newly adopted agriculture systems in the climate change driven agricultural frontiers work to mitigate carbon emission instead of contributing to it.

Food security was the least common dominant theme present in the literature, suggesting that this body of literature might not be well integrated with literature on agricultural systems and ecosystem services in the Himalayas specifically. There is a lack of quantitative research on the impacts of climate change on local food security in the Himalayas, especially in mountain regions. There is information on the challenges and current state of food security in some communities, but there is room to explore the positive impacts of climate change on local food availability through emerging CCDAFs in the Himalayas as this topic of research has not been discussed.

### Agricultural importance of main ecosystem services and impacts of climate change

The ecosystem services theme contained the largest number of sources. The most important ecosystem services mentioned in this literature are related to forests and water resources. The following section discusses interactions and significance of these ecosystem services to promote sustainable agriculture.

#### Himalayan forests and biodiversity

Forest degradation and biodiversity loss are products of both climate change and modern agricultural expansion, as well as various other anthropogenic pressures. One positive observation, however, is that climate change caused an upslope shift in treeline, altered species composition, and altered vegetation types resulting the increate in net primary productivity [4]. Contrary to this finding, Singh et al. [23] note that a significant increase in net primary productivity is not realistic in Himalayan forests. Quasim et al. [32] found that forests in high-altitude and mid-altitude zones of Pakistan have decreased by 30.5% and 49.7%, respectively, over 39 years. A third of this loss can be attributed to agricultural expansion [32]. Likewise, Qamer et al. [34] found that 170,684 ha of forest was lost or degraded in Pakistan from 1990 to 2010. In Mustang, Nepal, 42% of forest was lost from 1979 to 2009 largely as a result of agricultural expansion [1]. Similar trends in forest loss and degradation have also been seen in other parts of the Himalayas including Indian mountains and other mountain districts of Nepal [24, 35].

Forests in the Himalayan mountains provide important services that are crucial for the human well-being and ecosystem health. Mountain forests influence the quantity and quality of freshwater flows by capturing and storing snow and rain and reducing soil erosion [36], which is one of the leading causes behind increasing soil carbon emissions [35]. By contributing to soil stability and preventing soil loss and excess run-off, mountain forests help prevent landslides, avalanches, and floods [15]. Qamer et al. [34] explain that forest loss disrupts hydrological cycle which can lead to soil erosion, floods, and desertification in surrounding areas, further contributing to existing issues of water stress in local communities. Additionally, Himalayan forests play a crucial role in carbon sequestration. According to Singh [24], forests in the Indian Himalayas sequester approximately 65 million tonnes of carbon per year. Shrestha et al. [14] also found that forests contained higher soil organic carbon than other ecosystem types in Nepal. This ecosystem service is especially important in the context of climate change mitigation as it helps decrease atmospheric greenhouse gases. As forest ecosystems are converted to arable agriculture, the ability of the land to perform this important regulatory service is reduced, which implies that land sparing strategy of intensive agriculture to free up land for national parks and conservation areas. Hence, public policies should be in place to regulate agricultural expansion in the climate change driven agricultural frontiers in high altitudes with due consideration land sharing strategies so that we can avoid further release of greenhouse gasses from intensive agriculture.

Mountain forests also play a key role in human livelihoods by providing provisioning services. In the context of agriculture, Himalayan farmers depend on forests for feed and fodder for livestock, and manure from these livestock is used as fertilizer for crops [22]. Non timber forest products (NTFPs) are used for domestic and market purposes throughout the Himalayas and are especially important for remote communities in harsh mountain environments such as the Trans-Himalaya region [37]. NFTPs include fuel, construction materials, edible plants, and medicinal plants [37, 38]. Species richness of plants that provide NFTPs is lower at higher altitudes, however they are higher-value and provide a higher profit compared to NFTPs at lower altitudes. [37, 39]. This is especially true for medicinal plant harvests for local use as well as exports [37]. It is evident in the literature that mountain forests provide essential provisioning and regulatory services, and with agricultural expansion being a main cause of forest loss, it is important to understand the extent of these forests and quantify how they may be impacted by future agricultural expansion and whether land sharing strategies, such as agroforestry, are more important for agroecological transitions to low-carbon agricultural systems than land sparing through agricultural intensification.

Furthermore, high-altitude forests in the Himalayas typically contain higher biodiversity than lowland forests [36]. These areas provide important habitat for endemic species, which are particularly vulnerable to climate change. The added pressure of agricultural expansion and intensification puts biodiversity at further risk and may conflict with conservation efforts including the declaration of protected areas and the protection of endangered species [3, 40]. One study shows that future agriculture intensification in India, Nepal, and China may negatively impact biodiversity as these countries are rich in endemic species [30]. Maintaining biodiversity is an important part of mitigating climate change and building ecosystem resilience [41], however there is a lack of research on the specific impacts of agricultural expansion and intensification on biodiversity in this region.

The environmental and economic services outlined above are key contributors to human livelihood, agricultural productivity, and ecosystem function. There is a lack of quantifiable data on the potential extent of forest loss and degradation that could occur as a result of agricultural expansion in climate change driven agricultural frontiers, and the potential impacts to biodiversity loss and greenhouse gas emission in the Himalayas. It is evident that these impacts need to be better understood in order for effective land sharing and land sparing measures to be taken by governments and local communities in the Himalayas, especially as agricultural frontiers emerge in these areas.

#### Water security and Himalayan agriculture

Due to climate change, decreased water availability [12] has become a growing problem in higher altitude. Reduced snow fall, forward shifts in rainfall pattern, more front free days are common occurrences throughout the Himalayas although snow melt can increase short-term water availability in the lower altitudes [13]. A study by Pandey [42] shows that farmers in Upper Mustang, Nepal are experiencing decreased water availability and must get water from far away areas. Rice growers in India have had to change transplanting and harvesting time because of water scarcity [43]. In the context of agriculture, water stress is impacting crop productivity and income. With 90% of Himalayan agriculture being rain-fed, climate change poses a threat through decreased rainfall and increased drought frequency and intensity [23]. In some areas, precipitation is projected to increase but forward shifts in rainfall pattern can impact cropping patterns. This also poses challenges for farmers as agriculture in this region depends on water from snow melt, and increased rain paired with warmer temperatures cause snow to melt earlier and more quickly [23]. Farmers affected by these changes may experience lower crop yield and lower farm income. Moreover, arable cropland has higher rates of run-off and soil loss. As mentioned above, forests also play a crucial role in maintaining water quality, and loss of forests to agricultural expansion may result in increased sedimentation in streams [36].

Evidently, lack of water availability poses threats to rural communities and agricultural systems in the Himalayas which will be further exacerbated by changing precipitation trends and climate change. Decreasing water availability has been putting pressure on farmers along with the added stress of climate change and natural resource degradation [7]. However, there is a lack of quantifiable research on the state of water security in high-altitude communities in the Himalayas, and more specifically, the Trans-Himalaya region. It is also unclear how modern agricultural expansion in higher altitudes, such as replacing traditional crops with modern crop varieties, may impact water security. This information is important as water availability directly impacts agriculture, ecosystem function, and human livelihood in this region.

#### Ecosystem service impacts of agricultural adaptations to climate change

Agricultural adaptations were mentioned often in the literature that was grouped in the agriculture category. An extensive study on agricultural adaptations in the Hindu-Kush Himalayan region shows that farmers have noticed more frequent floods, droughts, landslides, pests, and diseases, and adapted their practices to these environmental changes through water conservation methods, changing sowing time, and introducing perianal crops that are more resilient to water stress and have high market value, such as temperate grape vines, fruits and nuts [21]. Farmers in India and the Loba community in Upper-Mustang, Nepal have noticed similar environmental changes and are adapting through crop diversification, increased vegetable and fruit farming, community involvement in labour, maintenance of irrigation systems, and migrating or abandoning cropland [42–44]. However, many of the introduced modern crop varieties for commercial farming are not necessarily the type of adaptations experts have recommended to promote agroecological transitions.

Additionally, agroforestry is mentioned often in the literature as an adaptation strategy that helps maintain ecosystem function as it stores carbon, contributes to biodiversity, prevents excess run-off and nutrient loss, increases soil organic matter and fertility, and maintains soil stability which helps prevent floods, landslides, and drought [5, 7, 45]. Multiple cropping has also been adopted as an effective adaptation strategy as it increases productivity, contributes to agrobiodiversity, and increases farm income [45, 46]. These methods are based on traditional ecological knowledge of local crop species and varieties [44]. Connections between traditional ecological knowledge, provisioning services, and cultural services are important to consider in the context of enabling agroecological transitions to low-carbon agricultural systems in the climate change driven Himalayan agricultural frontiers. As climate change poses new challenges in the region, neither traditional experiential knowledge nor expert scientific knowledge may be sufficient and farmers and scientists will have to engage in participatory research to co-create agricultural knowledge and technologies to help adapt to unpredictable and harsh climatic conditions in the high altitudes [7–47].

## Conclusion

The connections between climate change, agriculture, and ecosystem services are complex and play an important role in maintaining ecosystem functions and food security in the Himalayas. Majority of the reviewed literature were grouped under the ecosystem services theme, followed by agriculture and food security. A small portion of the literature had more than one dominant theme. It is evident that productivity of staple crops has been decreasing at lower altitudes in the Himalayas because of climate change, and farmers are noticing higher food insecurity and lower income as a result. With the threats of climate change, water stress, and food insecurity, agricultural adaptation is essential for the wellbeing of mountain communities without compromising the ecosystem services.

Mountain forests provide an extensive amount of ecosystem services including soil stabilization, water quality, carbon storage, biodiversity, and non-timber forest products. These services are crucial for ecosystem health and human wellbeing, especially at high-altitudes. Intensive agricultural expansion through the adoption of modern crop varieties at higher altitudes may decrease access to these important ecosystem services. With the general consensus being that land at higher altitudes may become suitable for crops as a result of climate change, the threat to forests and water resources will likely increase as agricultural expansion takes place. While the ecological and human value of forests and water security is well-understood in the literature, the extent by which they may be affected by agricultural expansion in the Himalayas is not known. This scoping review synthesises the evidence that gives us confidence to provide policy recommendations for sustainable agroecological transitions to low-carbon production systems. First, in order to increase crop yield and productivity, it is logical that farmers may take advantage of the newly available areas for agricultural expansion at higher altitudes. However, environmentally sound agricultural practices, such as agroforestry and perennial cropping must be implemented in the process to ensure long-term sustainability through agroecological transitions while protecting ecosystem functions. Public policies that conventionally promoted carbon-intensive industrial agriculture do not necessarily enable the adoption of agroecological transitions to low-carbon systems in the climate change driven agricultural frontiers. This scoping review has generated evidence that are credible enough to recommend that agricultural expansion in frontier areas in high mountains must be done through the adoption of land sharing technologies to preserve forest ecosystems that provide ecosystem services for the survival of high mountain civilizations. Second, since there is a very limited amount of quantifiable research on future crop suitability in the Himalayas, more research funding should be allocated to conduct agricultural frontier research that generate large scale quantitative data, including GIS and remote sensing. Frontier research benefits from long-term projections for 50 years or 100 years so that policy makers can consider research evidence to come up with proactive solutions to the problem of conventional focus on land sparing strategies. Specific research areas include the quantity and accessibility of land suitable for agricultural use in the frontier regions, specifically to promote agroecological transitions in climate change driven agricultural frontiers. Finally, governments in the Himalayan region should consider establishing an inter-governmental mechanism to facilitate regional collaboration to fund research on climate change driven agricultural frontiers. Such an inter-governmental body can learn from much advanced climate change driven agricultural frontier research in the North American and European Arctics.

## Electronic supplementary material

Below is the link to the electronic supplementary material.

Supplementary file1. Sources found from each keyword search using Web of Science and PubMed (DOCX 77KB).
