# Supplementary material for: Himalayan ecosystem services and climate change driven agricultural frontiers: a scoping review
Source: Discov Sustain. 2022 Oct 18;3(1):35. doi: 10.1007/s43621-022-00103-9 (PMC9579111; doi:10.1007/s43621-022-00103-9)
Supplement: Supplementary file 1 — Supplementary file1. Sources found from each keyword search using Web of Science and PubMed (DOCX 77KB). [file 43621_2022_103_MOESM1_ESM.docx]

Appendix A. Sources found from each keyword search using Web of Science and PubMed.

Sources found using Web of Science

1. Climate change AND Himalaya* AND Agriculture productivity Chhetri, & Easterling, W. E. (2010). Adapting to Climate Change: Retrospective Analysis of Climate Technology Interaction in the Rice-Based Farming System of Nepal. Annals of the Association of American Geographers, 100(5), 1156–1176. https://doi.org/10.1080/00045608.2010.518035
2. Vaidya, P. ., Randhawa, S. ., Sharma, P. ., Sharma, Y. P. ., Satyarthi, K. ., & Randhawa, S. S. . (2018). Climate variability and crop productivity in Himalayan ecosystem: A case study of Kullu district. MAUSAM, 69(4), 563–570. <https://doi.org/10.54302/mausam.v69i4.397>
3. Kamruzzaman, Rahman, A. T. M. S., Basak, A., Alam, J., & Das, J. (2022). Assessment and adaptation strategies of climate change through the prism of farmers’ perception: A case study. International Journal of Environmental Science and Technology (Tehran). https://doi.org/10.1007/s13762-022-04254-0
4. Bhatta, Udas, E., Khan, B., Ajmal, A., Amir, R., & Ranabhat, S. (2020). Local knowledge based perceptions on climate change and its impacts in the Rakaposhi valley of Gilgit-Baltistan, Pakistan. International Journal of Climate Change Strategies and Management, 12(2), 222–237. https://doi.org/10.1108/IJCCSM-05-2019-0024
5. Pratiksha Sharma, Rishi Ram Kattel, & Ananta Prakash Subedi. (2017). Climate change and maize agriculture among Chepang communities of Nepal: A review. Journal of Maize Research and Development, 3(1), 53–66.
6. Batool, N., Shah, S.A., Dar, S.N., Skinder, S. Rainfall variability and dynamics of cropping pattern in Kashmir Himalayas: a case study of climate change and agriculture. *SN Appl. Sci.* **1**, 606 (2019). <https://doi.org/10.1007/s42452-019-0599-9>
7. Gururani, K., Sood, S., Kumar, A., Joshi, D.C., Pandey, D., Sharma, A.R*.* (2021). Mainstreaming *Barahnaja* cultivation for food and nutritional security in the Himalayan region. *Biodivers Conserv* **30**, 551–574 (2021). <https://doi.org/10.1007/s10531-021-02123-9>
8. Khanal, Wilson, C., Hoang, V.-N., & Lee, B. (2018). Farmers’ Adaptation to Climate Change, Its Determinants and Impacts on Rice Yield in Nepal. Ecological Economics, 144, 139–147. https://doi.org/10.1016/j.ecolecon.2017.08.006
9. [Tiwari, PC](https://www.webofscience.com/wos/author/record/17404785)., [Joshi, B](https://www.webofscience.com/wos/author/record/23211964). Climate change and food security in Himalaya. [5th Symposium of Biodiversity and Natural Heritage of the Himalaya](https://www.webofscience.com/wos/woscc/general-summary?queryJson=%5B%7B%22rowBoolean%22:null,%22rowField%22:%22CF%22,%22rowText%22:%225th%20Symposium%20of%20Biodiversity%20and%20Natural%20Heritage%20of%20the%20Himalaya%22%7D%5D&eventMode=oneClickSearch). (2015)
10. Ramazan, & John, R. (2019). Climate Change Impacts and Mitigation; Abiotic Stress Impressions and Tolerance - A Perspective from Kashmir Himalayas. Proceedings of the Indian National Science Academy, Part A: Physical Sciences. https://doi.org/10.16943/ptinsa/2019/49676
11. Rymbai, D., Sheikh, F.M. The insight of agricultural adaptation to climate change: a case of rice growers in Eastern Himalaya, India. *Int J Biometeorol* **62**, 1833–1845 (2018). <https://doi.org/10.1007/s00484-018-1586-3>
12. Mishra, Rai, A., Abdelrahman, K., Rai, S. C., & Tiwari, A. (2021). Analysing Challenges and Strategies in Land Productivity in Sikkim Himalaya, India. *Sustainability (Basel, Switzerland)*, *13*(19), 11112–. <https://doi.org/10.3390/su131911112>
13. Rashid, I., Romshoo, S.A., Chaturvedi, R.K., Ravindranath, N.H., Sukumar, R., Jayaraman, M., Lakshmi, T.V., Sharma, J. Projected climate change impacts on vegetation distribution over Kashmir Himalayas. *Climatic Change* **132**, 601–613 (2015). <https://doi.org/10.1007/s10584-015-1456-5>
14. [Rayamajhi, N](https://www.webofscience.com/wos/author/record/27970534)., [Manandhar, B](https://www.webofscience.com/wos/author/record/3687109). Impact of climate change and adaptation measures on transhumance hearding system in Gatlang, Rasuwa. *Air, Soil and Water Research* **13**, (2020), <https://doi.org/10.1177/1178622120951173>
15. Ashraf, & Ahmad, I. (2021). Prospects of cryosphere-fed Kuhl irrigation system nurturing high mountain agriculture under changing climate in the Upper Indus Basin. The Science of the Total Environment, 788, 147752–147752. https://doi.org/10.1016/j.scitotenv.2021.147752
16. Tiwari, P.C., Joshi, B. Natural and socio-economic factors affecting food security in the Himalayas. *Food Sec.* 4, 195–207 (2012). https://doi.org/10.1007/s12571-012-0178-z
17. Rahut, & Ali, A. (2017). Coping with climate change and its impact on productivity, income, and poverty: Evidence from the Himalayan region of Pakistan. International Journal of Disaster Risk Reduction, 24, 515–525. https://doi.org/10.1016/j.ijdrr.2017.05.006
18. Rahut, & Ali, A. (2018). Impact of climate-change risk-coping strategies on livestock productivity and household welfare: empirical evidence from Pakistan. Heliyon, 4(10), e00797–e00797. https://doi.org/10.1016/j.heliyon.2018.e00797
19. Kumar, Jeena, N., Kumar, A., Khwairakpam, R., & Singh, H. (2021). Comparative response of rice cultivars to elevated air temperature in Bhabar region of Indian Himalaya: status on yield attributes. Heliyon, 7(7), e07474–e07474. https://doi.org/10.1016/j.heliyon.2021.e07474
20. Mondal, Srivastava, V. K., Roy, P. S., & Talukdar, G. (2014). Using logit model to identify the drivers of landuse landcover change in the Lower Gangetic Basin, India. International Archives of the Photogrammetry, Remote Sensing and Spatial Information Sciences., XL-8(8), 853–859. https://doi.org/10.5194/isprsarchives-XL-8-853-2014
21. Rajput, Bhardwaj, D. R., & Pala, N. A. (2016). Factors influencing biomass and carbon storage potential of different land use systems along an elevational gradient in temperate northwestern Himalaya. Agroforestry Systems, 91(3), 479–486. https://doi.org/10.1007/s10457-016-9948-5
22. Deb, Shrestha, S., & Babel, M. S. (2014). Forecasting climate change impacts and evaluation of adaptation options for maize cropping in the hilly terrain of Himalayas: Sikkim, India. Theoretical and Applied Climatology, 121(3-4), 649–667. https://doi.org/10.1007/s00704-014-1262-4
23. Iqbal, Akhter, G., Ashraf, A., & Ayub, S. (2018). Snowmelt runoff assessment and prediction under variable climate and glacier cover scenarios in Astore River Basin, Western Himalayas. Arabian Journal of Geosciences, 11(18), 1–8. https://doi.org/10.1007/s12517-018-3923-6
24. Khanal, Nepal, P., Zhang, Y., Nepal, G., Paudel, B., Liu, L., & Rai, R. (2020). Policy provisions for agricultural development in Nepal: A review. Journal of Cleaner Production, 261, 121241–. https://doi.org/10.1016/j.jclepro.2020.121241
25. Filippelli. (2008). The global phosphorus cycle; past, present, and future. Elements (Quebec), 4(2), 89–95. https://doi.org/10.2113/GSELEMENTS.4.2.89
26. Verma, A., Schmidt-Vogt, D., De Alban, J.D.T., Lim, C.L., Webb, E.L. Drivers and mechanisms of forest change in the Himalayas. *Global Environmental Change*. 68, 102-244 (2021). <https://doi.org/10.1016/j.gloenvcha.2021.102244>
27. Chatterjee, N. Soil erosion assessment in a humid, Eastern Himalayan watershed undergoing rapid land use changes, using RUSLE, GIS and high-resolution satellite imagery. *Model. Earth Syst. Environ.* 6, 533–543 (2020). <https://doi.org/10.1007/s40808-019-00700-0>
28. Rana, Singh, A., Sharma, Y., Pradheep, K., & Mendiratta, N. (2010). Dynamics of plant bioresources in Western Himalayan region of India – watershed based study. Current Science (Bangalore), 98(2), 192–203.
29. Arora, Bhatt, R., Sharma, V., & Hadda, M. S. (2022). Indigenous Practices of Soil and Water Conservation for Sustainable Hill Agriculture and Improving Livelihood Security. Environmental Management (New York). https://doi.org/10.1007/s00267-022-01602-1
30. Parmar B, Vishwakarma A, Padbhushan R, Kumar A, Kumar R, Kumari R, Kumar Yadav B, Giri SP, Kaviraj M and Kumar U (2022) Hedge and Alder-Based Agroforestry Systems: Potential Interventions to Carbon Sequestration and Better Crop Productivity in Indian Sub-Himalayas. *Front. Environ. Sci.* 10:858948. doi: 10.3389/fenvs.2022.858948
31. Martin, D., Lal, T., Sachdev, C.B., Sharma, J.P. Soil organic carbon storage changes with climate change, landform and land use conditions in Garhwal hills of the Indian Himalayan mountains. *Agriculture, Ecosystems & Environment*. **138**(1–2), 64-73. (2010). <https://doi.org/10.1016/j.agee.2010.04.001>.
32. Chauhan, Shukla, R., & Joshi, P. K. (2020). Assessing inherent vulnerability of farming communities across different biogeographical zones in Himachal Pradesh, India. Environmental Development, 33, 100506–. https://doi.org/10.1016/j.envdev.2020.100506
33. TSHEWANG, PARK, R. F., CHAUHAN, B. S., & JOSHI, A. K. (2018). CHALLENGES AND PROSPECTS OF WHEAT PRODUCTION IN BHUTAN: A REVIEW. Experimental Agriculture, 54(3), 428–442. https://doi.org/10.1017/S001447971700014X
34. Zahoor, S., Dutt, V., Mughal, A.H., Pala, N.A., Qaisar, K.N., Khan, P.A*.* Apple-based agroforestry systems for biomass production and carbon sequestration: implication for food security and climate change contemplates in temperate region of Northern Himalaya, India. *Agroforest Syst* **95**, 367–382 (2021). <https://doi.org/10.1007/s10457-021-00593-y>
35. Faisal, Abbas, A., Xia, C., Haseeb Raza, M., Akhtar, S., Arslan Ajmal, M., Mushtaq, Z., & Cai, Y. (2021). Assessing small livestock herders’ adaptation to climate variability and its impact on livestock losses and poverty. Climate Risk Management, 34, 100358–. https://doi.org/10.1016/j.crm.2021.100358
36. Kumar, & Sen, S. (2020). ASSESSMENT OF SPRING POTENTIAL FOR SUSTAINABLE AGRICULTURE: A CASE STUDY IN LESSER HIMALAYAS. Applied Engineering in Agriculture, 36(1), 11–24. <https://doi.org/10.13031/aea.13520>
37. Zheng, C., *et al.* Actual impacts of global warming on winter wheat yield in Eastern Himalayas. *International Journal of Plant Production.* **10**(2), 159-174. (2016)
38. Kumar, A., Dwivedi, G.K., Tewari, S., Paul, J., Anand, R., Kumar, N., Kumar, P., Singh, H., Kaushal, R. Carbon Mineralization and Inorganic Nitrogen Pools under *Terminalia chebula* Retz.-Based Agroforestry System in Himalayan Foothills, India. *Forest Science*, **66**(5), 634–643. (2020). <https://doi.org/10.1093/forsci/fxaa012>
39. Shukla, Sachdeva, K., & Joshi, P. K. (2016). Inherent vulnerability of agricultural communities in Himalaya: A village-level hotspot analysis in the Uttarakhand state of India. Applied Geography (Sevenoaks), 74, 182–198. https://doi.org/10.1016/j.apgeog.2016.07.013
40. Choudhury, Fiyaz, A. R., Mohapatra, K. P., & Ngachan, S. (2016). Impact of Land Uses, Agrophysical Variables and Altitudinal Gradient on Soil Organic Carbon Concentration of North-Eastern Himalayan Region of India. Land Degradation & Development, 27(4), 1163–1174. https://doi.org/10.1002/ldr.2338
41. Choudhary, Panday, S. C., Meena, V. S., Singh, S., Yadav, R. P., Pattanayak, A., Mahanta, D., Bisht, J. K., & Stanley, J. (2020). Long-term tillage and irrigation management practices: Strategies to enhance crop and water productivity under rice-wheat rotation of Indian mid-Himalayan Region. Agricultural Water Management, 232, 106067–. <https://doi.org/10.1016/j.agwat.2020.106067>
42. Hajong, S., *et al.* Genotypic variability and physio-morphological efficiency of buckwheat (Fagopyrum spp.) under moisture stress at mid-altitudes of Meghalaya (India). *Crop and Pasture Science.* (2022). DOI10.1071/CP22062
43. Das, Rangappa, K., Basavaraj, S., Dey, U., Haloi, M., Layek, J., Idapuganti, R. G., Lal, R., Deshmukh, N. A., Yadav, G. S., Babu, S., & Ngachan, S. (2021). Conservation tillage and nutrient management practices in summer rice (Oryza sativa L.) favoured root growth and phenotypic plasticity of succeeding winter pea (Pisum sativumL.) under eastern Himalayas, India. Heliyon, 7(5), e07078–e07078. https://doi.org/10.1016/j.heliyon.2021.e07078
44. Bhattacharyya, Bhatia, A., Das, T. K., Lata, S., Kumar, A., Tomer, R., Singh, G., Kumar, S., & Biswas, A. K. (2018). Aggregate-associated N and global warming potential of conservation agriculture-based cropping of maize-wheat system in the north-western Indo-Gangetic Plains. Soil & Tillage Research, 182, 66–77. https://doi.org/10.1016/j.still.2018.05.002

Climate change AND Himalaya* AND Crop yield

1. Batool, N., Shah, S.A., Dar, S.N., Skinder, S. Rainfall variability and dynamics of cropping pattern in Kashmir Himalayas: a case study of climate change and agriculture. *SN Appl. Sci.* **1**, 606 (2019). <https://doi.org/10.1007/s42452-019-0599-9>
2. Palazzoli, I., Maskey, S., Uhlenbrook, S., Nana, E., Bocchiola, D. Impact of prospective climate change on water resources and crop yields in the Indrawati basin, Nepal. *Agricultural Systems*. **133**, 143-157 (2015). DOI: 10.1016/j.agsy.2014.10.016
3. Acharya. (2018). The Effects of Changing Climate and Market Conditions on Crop Yield and Acreage Allocation in Nepal. Climate (Basel), 6(2), 32–. https://doi.org/10.3390/cli6020032
4. Daloz, Rydsaa, J. H., Hodnebrog, Ø., Sillmann, J., van Oort, B., Mohr, C. W., Agrawal, M., Emberson, L., Stordal, F., & Zhang, T. (2021). Direct and indirect impacts of climate change on wheat yield in the Indo-Gangetic plain in India. Journal of Agriculture and Food Research, 4, 100132–. https://doi.org/10.1016/j.jafr.2021.100132
5. Das, J., Poonia, V., Jha, S., Goyal, M.K*.* Understanding the climate change impact on crop yield over Eastern Himalayan Region: ascertaining GCM and scenario uncertainty. *Theor Appl Climatol* **142**, 467–482 (2020). https://doi.org/10.1007/s00704-020-03332-y
6. Deb, Shrestha, S., & Babel, M. S. (2014). Forecasting climate change impacts and evaluation of adaptation options for maize cropping in the hilly terrain of Himalayas: Sikkim, India. Theoretical and Applied Climatology, 121(3-4), 649–667. https://doi.org/10.1007/s00704-014-1262-4
7. Bocchiola, Brunetti, L., Soncini, A., Polinelli, F., & Gianinetto, M. (2019). Impact of climate change on agricultural productivity and food security in the Himalayas: A case study in Nepal. Agricultural Systems, 171, 113–125. https://doi.org/10.1016/j.agsy.2019.01.008
8. Rana, R.S., *et al.* (2018). Study on maize production under changed climatic scenarios in western Himalaya of India. Mausam. 69(2), 323-330.
9. Rawat, P.K. Impacts of climate change and hydrological hazards on monsoon crop patterns in the Lesser Himalaya: A watershed based study. *Int J Disaster Risk Sci* **3**, 98–112 (2012). https://doi.org/10.1007/s13753-012-0010-6
10. Bhattarai, Beilin, R., & Ford, R. (2015). Gender, Agrobiodiversity, and Climate Change: A Study of Adaptation Practices in the Nepal Himalayas. World Development, 70, 122–132. https://doi.org/10.1016/j.worlddev.2015.01.003
11. Rawat. (2014). GIS development to monitor climate change and its geohydrological consequences on non-monsoon crop pattern in Himalaya. Computers & Geosciences, 70, 80–95. https://doi.org/10.1016/j.cageo.2014.04.010
12. Muslim, M., Romshoo, S.A. & Rather, A.Q. Paddy crop yield estimation in Kashmir Himalayan rice bowl using remote sensing and simulation model. *Environ Monit Assess* **187**, 316 (2015). https://doi.org/10.1007/s10661-015-4564-9
13. Meena, R.K. (2019). Local perceptions and adaptation of indigenous communities to climate change: Evidences from High Mountain Pangi valley of Indian Himalayas. *Indian Journal of Traditional Knowledge.* 18(1), 58-67.
14. Pandey, R., Aretano, R., Gupta, A.K., Meena, D., Kumar, B., Alatalo, J.M*.* Agroecology as a Climate Change Adaptation Strategy for Smallholders of Tehri-Garhwal in the Indian Himalayan Region. *Small-scale Forestry* **16**, 53–63 (2017). https://doi.org/10.1007/s11842-016-9342-1
15. [Mahajan, V](https://www.webofscience.com/wos/author/record/15277222)., Singh, K.P., [Rajendran, R.A](https://www.webofscience.com/wos/author/record/27129942)., [Kanya](https://www.webofscience.com/wos/author/record/9723156). Response of maize genotypes to changing climatic conditions in Himalayan region. *Indian Journal of Genetics and Plant Breeding*. **72**(2), 183-188. (2012).
16. [Rana, JC](https://www.webofscience.com/wos/author/record/27981027), [Sharma, SK](https://www.webofscience.com/wos/author/record/14259170). Plant genetic resources management under emerging climate change. *Indian Journal of Genetics and Plant Breeding*. 69(4), 267-283. (2009)
17. Datta, P., Behera, B. What caused smallholders to change farming practices in the era of climate change? Empirical evidence from Sub-Himalayan West Bengal, India. *GeoJournal* (2021). <https://doi.org/10.1007/s10708-021-10450-1>
18. Sharma, R.K., Shrestha, D.G. Climate perceptions of local communities validated through scientific signals in Sikkim Himalaya, India. *Environ Monit Assess* **188**, 578 (2016). https://doi.org/10.1007/s10661-016-5582-y
19. Poudel, Funakawa, S., & Shinjo, H. (2017). Household Perceptions about the Impacts of Climate Change on Food Security in the Mountainous Region of Nepal. Sustainability (Basel, Switzerland), 9(4), 641–. https://doi.org/10.3390/su9040641
20. Dahal, N.M. Factors affecting maize, rice and wheat yields in the Koshi River Basin, Nepal. *Journal of Agriculture Meteorology.* 77(3),179-189. (2021). <https://doi.org/10.2480/agrmet.D-20-00019>
21. Deb, Kiem, A. S., Babel, M. S., Chu, S. T., & Chakma, B. (2015). Evaluation of climate change impacts and adaptation strategies for maize cultivation in the Himalayan foothills of India. *Journal of Water and Climate Change*, *6*(3), 596–614. <https://doi.org/10.2166/wcc.2015.070>
22. Poonia, Das, J., & Goyal, M. K. (2021). Impact of climate change on crop water and irrigation requirements over eastern Himalayan region. Stochastic Environmental Research and Risk Assessment, 35(6), 1175–1188. https://doi.org/10.1007/s00477-020-01942-6
23. Shakoor, Sofi, N. R., Hussain, A., Khan, G. H., Sofi, M., Mohiddin, F. A., Wani, S. H., Mehdi, S. S., Bhat, N. A., & Shikari, A. B. (2022). Crop simulation mediated assessment of climate change impact on rice grown under temperate high-altitude valley of Kashmir. *Theoretical and Applied Climatology*, *147*(3-4), 1437–1451. <https://doi.org/10.1007/s00704-021-03880-x>
24. Pratiksha Sharma, Rishi Ram Kattel, & Ananta Prakash Subedi. (2017). Climate change and maize agriculture among Chepang communities of Nepal: A review. Journal of Maize Research and Development, 3(1), 53–66.
25. Zheng, C., *et al.* Actual impacts of global warming on winter wheat yield in Eastern Himalayas. *International Journal of Plant Production.* **10**(2), 159-174. (2016)
26. Ranjan. (2021). Land use decisions under REDD plus incentives when warming temperatures affect crop productivity and forest biomass growth rates. Land Use Policy, 108. https://doi.org/10.1016/j.landusepol.2021.105595
27. Tripathi, P., Shah, S., Kashyap, S.D., Tripathi, A*.* Fruit yield and quality characteristics of high density *Prunus persica* (L.) Batsch plantation intercropped with medicinal and aromatic plants in the Indian Western Himalayas. *Agroforest Syst* **93**, 1717–1728 (2019). <https://doi.org/10.1007/s10457-018-0276-9>
28. Kumar, Jeena, N., Kumar, A., Khwairakpam, R., & Singh, H. (2021). Comparative response of rice cultivars to elevated air temperature in Bhabar region of Indian Himalaya: status on yield attributes. Heliyon, 7(7), e07474–e07474. https://doi.org/10.1016/j.heliyon.2021.e07474
29. Wassmann, Jagadish, S. V. K., Sumfleth, K., Pathak, H., Howell, G., Ismail, A., Serraj, R., Redona, E., Singh, R. K., & Heuer, S. (2009). Chapter 3 Regional Vulnerability of Climate Change Impacts on Asian Rice Production and Scope for Adaptation. In *Advances in Agronomy* (Vol. 102, pp. 91–133). Elsevier Science & Technology. <https://doi.org/10.1016/S0065-2113(09)01003-7>
30. Umesh Babu, Saha, L., & Garkoti, S. C. (2020). Changing socio-economic and climate scenario calls for documentation of the traditional knowledge and practices related to riverbed cultivation: a case study of a migrant farming community from Western Himalaya, India. Agroecology and Sustainable Food Systems, 44(3), 310–330. https://doi.org/10.1080/21683565.2019.1622618
31. McDowell, Ford, J. D., Lehner, B., Berrang-Ford, L., & Sherpa, A. (2012). Climate-related hydrological change and human vulnerability in remote mountain regions: a case study from Khumbu, Nepal. Regional Environmental Change, 13(2), 299–310. https://doi.org/10.1007/s10113-012-0333-2
32. Wagle, Dhakal, M. P., & Shrestha, A. B. (2021). Adaptation Strategies to Address Challenges of Traditional Agricultural Water Management in the Upper Indus Basin. Mountain Research and Development, 41(3), R24–R31. https://doi.org/10.1659/MRD-JOURNAL-D-20-00059.1
33. Biemans, Siderius, C., Mishra, A., & Ahmad, B. (2016). Crop-specific seasonal estimates of irrigation-water demand in South Asia. *Hydrology and Earth System Sciences*, *20*(5), 1971–1982. <https://doi.org/10.5194/hess-20-1971-2016>
34. Panjwani, S., Naresh Kumar, S., Ahuja, L., Islam, A*.* Evaluation of selected global climate models for extreme temperature events over India. *Theor Appl Climatol* **140**, 731–738 (2020). <https://doi.org/10.1007/s00704-020-03108-4>
35. Misra. (2012). Climate change impact, mitigation and adaptation strategies for agricultural and water resources, in Ganga Plain (India). Mitigation and Adaptation Strategies for Global Change, 18(5), 673–689. https://doi.org/10.1007/s11027-012-9381-7
36. Rahut, & Ali, A. (2017). Coping with climate change and its impact on productivity, income, and poverty: Evidence from the Himalayan region of Pakistan. International Journal of Disaster Risk Reduction, 24, 515–525. https://doi.org/10.1016/j.ijdrr.2017.05.006
37. Luitel, Siwakoti, M., Joshi, M. D., Rangaswami, M., & Jha, P. K. (2020). Potential suitable habitat of Eleusine coracana (L) gaertn (Finger millet) under the climate change scenarios in Nepal. BMC Ecology, 20(1), 19–19. https://doi.org/10.1186/s12898-020-00287-6
38. Forsythe, N., Blenkinsop, S., and Fowler, H. J.: Exploring objective climate classification for the Himalayan arc and adjacent regions using gridded data sources, Earth Syst. Dynam., 6, 311–326, (2015). https://doi.org/10.5194/esd-6-311-2015
39. Kumar, & Sen, S. (2020). ASSESSMENT OF SPRING POTENTIAL FOR SUSTAINABLE AGRICULTURE: A CASE STUDY IN LESSER HIMALAYAS. Applied Engineering in Agriculture, 36(1), 11–24. https://doi.org/10.13031/aea.13520
40. Babel, Deb, P., & Soni, P. (2018). Performance Evaluation of AquaCrop and DSSAT-CERES for Maize Under Different Irrigation and Manure Application Rates in the Himalayan Region of India. Agricultural Research (India : Online), 8(2), 207–217. https://doi.org/10.1007/s40003-018-0366-y
41. Krupnik, Timsina, J., Devkota, K. P., Tripathi, B. P., Karki, T. B., Urfels, A., Gaihre, Y. K., Choudhary, D., Beshir, A. R., Pandey, V. P., Brown, B., Gartaula, H., Shahrin, S., & Ghimire, Y. N. (2021). Agronomic, socio-economic, and environmental challenges and opportunities in Nepal’s cereal-based farming systems (Vol. 170, pp. 155–287). Elsevier. https://doi.org/10.1016/bs.agron.2021.06.004
42. Ramirez-Villegas, & Challinor, A. (2012). Assessing relevant climate data for agricultural applications. Agricultural and Forest Meteorology, 161, 26–45. https://doi.org/10.1016/j.agrformet.2012.03.015
43. Dad, Dand, S. A., & Pala, N. A. (2021). The effect of bi-culture cover crops on soil quality, carbon [sequestration, and growth characteristics](sequestration,%20and%20growth%20characteristics) in apple orchards of North Western Himalayas. Agroforestry Systems, 95(8), 1745–1758. <https://doi.org/10.1007/s10457-021-00687-7>
44. Baiysha, LK., *et al.* Crop and varietal diversification for enhancing productivity and profitability of rice fallow system in eastern Himalayan region. *Indian Journal of Agricultural Sciences.* 89(5), 800-805. (2019)
45. Pal, & Mahajan, M. (2017). Tillage system and organic mulch influence leaf biomass, steviol glycoside yield and soil health under sub-temperate conditions. Industrial Crops and Products, 104, 33–44. https://doi.org/10.1016/j.indcrop.2017.04.012
46. Hajong, S., *et al.* Genotypic variability and physio-morphological efficiency of buckwheat (Fagopyrum spp.) under moisture stress at mid-altitudes of Meghalaya (India). *Crop and Pasture Science.* (2022). DOI10.1071/CP22062
47. Wu, Xu, M., Peng, Z., & Chen, X. (2022). Quantifying the potential impacts of meltwater on cotton yields in the Tarim River Basin, Central Asia. Agricultural Water Management, 269, 107639–. https://doi.org/10.1016/j.agwat.2022.107639
48. Choudhary, Panday, S. C., Meena, V. S., Singh, S., Yadav, R. P., Pattanayak, A., Mahanta, D., Bisht, J. K., & Stanley, J. (2020). Long-term tillage and irrigation management practices: Strategies to enhance crop and water productivity under rice-wheat rotation of Indian mid-Himalayan Region. Agricultural Water Management, 232, 106067–. <https://doi.org/10.1016/j.agwat.2020.106067>
49. Wong, KV., Chadhry, S. (2015). Climate change aggravates the energy-water-food nexus. *IMECE* Vol 6A
50. Gupta, Pathania, P., Bala, I., & Sood, P. (2016). Assessment of genetic variation, diversity, and resistance to Helicoverpa armigera in cultivated chickpea (Cicer arietinum L.) undernew agro-climatic zone. *Legume Research*, *OF*. <https://doi.org/10.18805/lr.v0iOF.11049>
51. Mäder, Kaiser, F., Adholeya, A., Singh, R., Uppal, H. S., Sharma, A. K., Srivastava, R., Sahai, V., Aragno, M., Wiemken, A., Johri, B. N., & Fried, P. M. (2011). Inoculation of root microorganisms for sustainable wheat–rice and wheat–black gram rotations in India. Soil Biology & Biochemistry, 43(3), 609–619. https://doi.org/10.1016/j.soilbio.2010.11.031
52. Choudhury, Nengzouzam, G., & Islam, A. (2022). Runoff and soil erosion in the integrated farming systems based on micro-watersheds under projected climate change scenarios and adaptation strategies in the eastern Himalayan mountain ecosystem (India). Journal of Environmental Management, 309, 114667–114667. https://doi.org/10.1016/j.jenvman.2022.114667
53. TSHEWANG, PARK, R. F., CHAUHAN, B. S., & JOSHI, A. K. (2018). CHALLENGES AND PROSPECTS OF WHEAT PRODUCTION IN BHUTAN: A REVIEW. Experimental Agriculture, 54(3), 428–442. https://doi.org/10.1017/S001447971700014X
54. Das, Rangappa, K., Basavaraj, S., Dey, U., Haloi, M., Layek, J., Idapuganti, R. G., Lal, R., Deshmukh, N. A., Yadav, G. S., Babu, S., & Ngachan, S. (2021). Conservation tillage and nutrient management practices in summer rice (Oryza sativa L.) favoured root growth and phenotypic plasticity of succeeding winter pea (Pisum sativumL.) under eastern Himalayas, India. Heliyon, 7(5), e07078–e07078. <https://doi.org/10.1016/j.heliyon.2021.e07078>
55. Ghimire, K.H., Joshi, B.K., Gurung, R., Sthapit, B.R*.* Nepalese foxtail millet [*Setaria italica* (L.) P. Beauv.] genetic diversity revealed by morphological markers. *Genet Resour Crop Evol* **65**, 1147–1157 (2018). https://doi.org/10.1007/s10722-017-0602-5
56. Khatiwada, & Pandey, V. P. (2019). Characterization of hydro-meteorological drought in Nepal Himalaya: A case of Karnali River Basin. Weather and Climate Extremes, 26, 100239–. https://doi.org/10.1016/j.wace.2019.100239
57. Kumar, Murugan, P., Murkute, A., & Bala Singh, Sh. (2010). A carbon sequestration strategy involving temperate fruit crops in the trans-Himalayan region. The Journal of Horticultural Science & Biotechnology, 85(5), 405–409. https://doi.org/10.1080/14620316.2010.11512688x
58. Yaseen, Latif, Y., Waseem, M., Leta, M., Abbas, S., & Akram Bhatti, H. (2022). Contemporary Trends in High and Low River Flows in Upper Indus Basin, Pakistan. Water (Basel), 14(3), 337–. https://doi.org/10.3390/w14030337
59. [Angus, S.D](https://www.webofscience.com/wos/author/record/50692)., [Parris, B](https://www.webofscience.com/wos/author/record/12635537)., [Hassani-M, B](https://www.webofscience.com/wos/author/record/930906). Climate change impacts and adaptation in Bangladesh: An agent-based approach. [18th MODSIM09 Biennial Conference on Modelling and Simulation](https://www.webofscience.com/wos/woscc/general-summary?queryJson=%5B%7B%22rowBoolean%22:null,%22rowField%22:%22CF%22,%22rowText%22:%22IMACS%20World%20Congress%2FModelling%20and%20Simulation%20Society-of-Australia-and-New-Zealand%20(MSSANZ)%2F18th%20MODSIM09%20Biennial%20Conference%20on%20Modelling%20and%20Simulation%22%7D%5D&eventMode=oneClickSearch). (2009)
60. Joship, BR., Mahato, SN. (2013). Gastrointestinal Parasitic Diseases of Buffaloes and Implications of Climate Change for these Diseases in Nepal. Buffalo Bulletin. 32, 1082-1087.
61. Yadav, Das, A., Lal, R., Babu, S., Datta, M., Meena, R. S., Patil, S. B., & Singh, R. (2019). Impact of no-till and mulching on soil carbon sequestration under rice (Oryza sativa L.)-rapeseed (Brassica campestris L. var. rapeseed) cropping system in hilly agro-ecosystem of the Eastern Himalayas, India. Agriculture, Ecosystems & Environment, 275, 81–92. https://doi.org/10.1016/j.agee.2019.02.001
62. Raghuvanshi, & Sharma, R. K. (2016). Response of two cultivars of Phaseolus vulgaris L. (French beans) plants exposed to enhanced UV-B radiation under mountain ecosystem. Environmental Science and Pollution Research International, 23(1), 831–842. https://doi.org/10.1007/s11356-015-5332-7
63. Sharma, Walia, S., Rathore, S., Kumar, P., & Kumar, R. (2020). Combined effect of elevated CO2 and temperature on growth, biomass and secondary metabolite of Hypericum perforatum L. in a western Himalayan region. Journal of Applied Research on Medicinal and Aromatic Plants, 16, 100239–. https://doi.org/10.1016/j.jarmap.2019.100239
64. Putero, Marinoni, A., Calzolari, F., Rupakheti, M., Cristofanelli, P., & Bonasoni, P. (2018). Black Carbon and Ozone Variability at the Kathmandu Valley and at the Southern Himalayas: A Comparison between a ＂Hot Spot＂ and a Downwind High-Altitude Site. Aerosol and Air Quality Research, 18(3), 623–635. https://doi.org/10.4209/aaqr.2017.04.0138
65. Das, Layek, J., Ramkrushna, G. I., Rangappa, K., Lal, R., Ghosh, P. K., Choudhury, B. U., Mandal, S., Ngangom, B., Dey, U., & Prakash, N. (2019). Effects of tillage and rice residue management practices on lentil root architecture, productivity and soil properties in India’s Lower Himalayas. Soil & Tillage Research, 194, 104313–. https://doi.org/10.1016/j.still.2019.104313
66. Nagar, Rawat, S., Pandey, R., Kumar, M., & Alatalo, J. M. (2021). Fuelwood and fodder consumption patterns among agroforestry-practicing smallholder farmers of the lower Himalayas, India. Environment, Development and Sustainability, 24(4), 5594–5613. <https://doi.org/10.1007/s10668-021-01673-w>
67. Choudhury, Nengzouzam, G., Mandal, S., Sethy, B. K., Hazarika, S., & Mishra, V. K. (2022). Long‐term effect of integrated farming systems on soil erosion in hilly micro‐watersheds (Indian Eastern Himalayas). Land Degradation & Development, 33(14), 2554–2566. https://doi.org/10.1002/ldr.4332
68. Leipe, Demske, D., Tarasov, P. E., Wünnemann, B., & Riedel, F. (2014). Potential of pollen and non-pollen palynomorph records from Tso Moriri (Trans-Himalaya, NW India) for reconstructing Holocene limnology and human–environmental interactions. Quaternary International, 348, 113–129. https://doi.org/10.1016/j.quaint.2014.02.026
69. Kumar, Dwivedi, G. K., Tewari, S., Paul, J., Anand, R., Kumar, N., Kumar, P., Singh, H., & Kaushal, R. (2020). Carbon Mineralization and Inorganic Nitrogen Pools under Terminalia chebula Retz.-Based Agroforestry System in Himalayan Foothills, India. Forest Science, 66(5), 634–643. https://doi.org/10.1093/forsci/fxaa012
70. Golombek, & Blanke, M. (2020). Apple Supply Chain in India-From The Himalayan Mountains to The Consumer. Erwerbsobstbau, 62(4), 377–387. https://doi.org/10.1007/s10341-020-00515-9

Climate change AND Himalaya* AND Crop suitability

1. Heider, K., Weinzierl, T., Schwab, N., Bobrowski, M., & Schickhoff, U. (2018). Future agricultural conditions in the Nepal Himalaya - A fuzzy logic approach using high resolution climate scenarios. *J. Geogr. Soc. Berl*. 149(4), 227-240. DOI:10.12854/erde-2018-382
2. Luitel, Siwakoti, M., Joshi, M. D., Rangaswami, M., & Jha, P. K. (2020). Potential suitable habitat of Eleusine coracana (L) gaertn (Finger millet) under the climate change scenarios in Nepal. BMC Ecology, 20(1), 19–19. https://doi.org/10.1186/s12898-020-00287-6
3. Manandhar, Pandey, V. P., & Kazama, F. (2014). Assessing suitability of apple cultivation under climate change in mountainous regions of western Nepal. Regional Environmental Change, 14(2), 743–756. <https://doi.org/10.1007/s10113-013-0531-6>
4. [Rana, JC](https://www.webofscience.com/wos/author/record/27981027), [Sharma, SK](https://www.webofscience.com/wos/author/record/14259170). Plant genetic resources management under emerging climate change. *Indian Journal of Genetics and Plant Breeding*. 69(4), 267-283. (2009)
5. Negi, Maikhuri, R. K., Chandra, A., Maletha, A., & Dhyani, P. P. (2018). Assessing sustainability of farming systems in mountain agroecosystems of Western Himalaya, India. Agroecology and Sustainable Food Systems, 42(7), 751–776. <https://doi.org/10.1080/21683565.2018.1427175>
6. Ashraf A, Ahmad I. Prospects of cryosphere-fed Kuhl irrigation system nurturing high mountain agriculture under changing climate in the Upper Indus Basin. Sci Total Environ. 2021 Sep 20;788:147752. doi: 10.1016/j.scitotenv.2021.147752. Epub 2021 May 14. PMID: 34020094.
7. Deb, Debnath, P., Denis, A. F., & Lepcha, O. T. (2018). Variability of soil physicochemical properties at different agroecological zones of Himalayan region: Sikkim, India. Environment, Development and Sustainability, 21(5), 2321–2339. https://doi.org/10.1007/s10668-018-0137-8
8. Sharma, S., Arunachalam, K. & Arunachalam, A. Morphology and physiology of *Perilla frutescens* (Linn.) Britt in relation to micro-climate and edaphic characteristics. *Trop Ecol* **63**, 375–386 (2022). https://doi.org/10.1007/s42965-021-00195-w

Climate change AND Himalaya* AND Ecosystem service*

1. Palomo. (2017). Climate Change Impacts on Ecosystem Services in High Mountain Areas: A Literature Review. Mountain Research and Development, 37(2), 179–187. <https://doi.org/10.1659/MRD-JOURNAL-D-16-00110.1>
2. Khan, Sharma, A., & Goyal, M. K. (2019). Assessment of future water provisioning and sediment load under climate and LULC change scenarios in a peninsular river basin, India. Hydrological Sciences Journal, 64(4), 405–419. https://doi.org/10.1080/02626667.2019.1584401
3. Aukema, Pricope, N. G., Husak, G. J., & Lopez-Carr, D. (2017). Biodiversity Areas under Threat: Overlap of Climate Change and Population Pressures on the World’s Biodiversity Priorities. PloS One, 12(1), e0170615–e0170615. https://doi.org/10.1371/journal.pone.0170615
4. Kattel. (2022). Climate warming in the Himalayas threatens biodiversity, ecosystem functioning and ecosystem services in the 21st century: is there a better solution? Biodiversity and Conservation, 31(8-9), 2017–2044. https://doi.org/10.1007/s10531-022-02417-6
5. Negi, Maikhuri, R. K., Pharswan, D., Thakur, S., & Dhyani, P. P. (2017). Climate change impact in the Western Himalaya: people’s perception and adaptive strategies. Journal of Mountain Science, 14(2), 403–416. https://doi.org/10.1007/s11629-015-3814-1
6. Pandey, Aretano, R., Gupta, A. K., Meena, D., Kumar, B., & Alatalo, J. M. (2016). Agroecology as a Climate Change Adaptation Strategy for Smallholders of Tehri-Garhwal in the Indian Himalayan Region. Small-Scale Forestry, 16(1), 53–63. https://doi.org/10.1007/s11842-016-9342-1
7. Saeed, Arshad, M., Hayat, S., Morelli, T. L., & Ali Nawaz, M. (2022). Analysis of provisioning ecosystem services and perceptions of climate change for indigenous communities in the Western Himalayan Gurez Valley, Pakistan. Ecosystem Services, 56. https://doi.org/10.1016/j.ecoser.2022.101453
8. Tewari, Verma, R. K., & von Gadow, K. (2017). Climate change effects in the Western Himalayan ecosystems of India: evidence and strategies. Forest Ecosystems, 4(1), 1–9. https://doi.org/10.1186/s40663-017-0100-4
9. Mills, Tan, D., Manji, A. K., Vijitpan, T., Henriette, E., Murugaiyan, P., Pantha, R. H., Lafdal, M. Y., Soule, A., Cazzetta, S., Bégat, P., Vlieghe, K. E. P., Lavirotte, L., Kok, J. T., & Lister, J. (2020). Ecosystem‐based adaptation to climate change: Lessons learned from a pioneering project spanning Mauritania, Nepal, the Seychelles, and China. Plants, People, Planet, 2(6), 587–597. https://doi.org/10.1002/ppp3.10126
10. Singh, & Thadani, R. (2015). Complexities and Controversies in Himalayan Research: A Call for Collaboration and Rigor for Better Data. Mountain Research and Development, 35(4), 401–409. https://doi.org/10.1659/MRD-JOURNAL-D-15-00045
11. Lamsal, Kumar, L., Aryal, A., & Atreya, K. (2018). Invasive alien plant species dynamics in the Himalayan region under climate change. Ambio, 47(6), 697–710. https://doi.org/10.1007/s13280-018-1017-z
12. Negi, Pathak, R., Rawal, R. S., Bhatt, I. D., & Sharma, S. (2019). Long-term ecological monitoring on forest ecosystems in Indian Himalayan Region: Criteria and indicator approach. Ecological Indicators, 102, 374–381. https://doi.org/10.1016/j.ecolind.2019.02.035
13. Karki, Bhatta, B., Devkota, N. R., Acharya, R. P., & Kunwar, R. M. (2021). Climate Change Adaptation (CCA) Interventions and Indicators in Nepal: Implications for Sustainable Adaptation. Sustainability (Basel, Switzerland), 13(23), 13195–. https://doi.org/10.3390/su132313195
14. Shirmohammadi, Malekian, A., Salajegheh, A., Taheri, B., Azarnivand, H., Malek, Z., & Verburg, P. H. (2020). Impacts of future climate and land use change on water yield in a semiarid basin in Iran. Land Degradation & Development, 31(10), 1252–1264. https://doi.org/10.1002/ldr.3554
15. Mondal, & Zhang, Y. (2018). Research Progress on Changes in Land Use and Land Cover in the Western Himalayas (India) and Effects on Ecosystem Services. Sustainability (Basel, Switzerland), 10(12), 4504–. https://doi.org/10.3390/su10124504
16. Shahzad, Tahir, A., Sharif, F., Haq, I. U., & Mukhtar, H. (2019). Assessing the impacts of changing climate on forest ecosystem services and livelihood of Balakot mountainous communities. Pakistan Journal of Botany, 51(4). https://doi.org/10.30848/PJB2019-4(1)
17. Momblanch, Beevers, L., Srinivasalu, P., Kulkarni, A., & Holman, I. P. (2020). Enhancing production and flow of freshwater ecosystem services in a managed Himalayan river system under uncertain future climate. Climatic Change, 162(2), 343–361. https://doi.org/10.1007/s10584-020-02795-2
18. Xu, Sharma, R., Fang, J., & Xu, Y. (2008). Critical linkages between land-use transition and human health in the Himalayan region. Environment International, 34(2), 239–247. https://doi.org/10.1016/j.envint.2007.08.004
19. Kumar, Savita, Singh, H., Pandey, R., Singh, M. P., Ravindranath, N. H., & Kalra, N. (2018). Assessing vulnerability of forest ecosystem in the Indian Western Himalayan region using trends of net primary productivity. Biodiversity and Conservation, 28(8-9), 2163–2182. https://doi.org/10.1007/s10531-018-1663-2
20. Shah, Sharma, S., Haase, P., Jähnig, S. C., & Pauls, S. U. (2015). The climate sensitive zone along an altitudinal gradient in central Himalayan rivers: a useful concept to monitor climate change impacts in mountain regions. Climatic Change, 132(2), 265–278. https://doi.org/10.1007/s10584-015-1417-z
21. Gurung, Miller, K. K., Venn, S., & Bryan, B. A. (2021). Climate change adaptation for managing non-timber forest products in the Nepalese Himalaya. The Science of the Total Environment, 796, 148853–148853. https://doi.org/10.1016/j.scitotenv.2021.148853
22. van Oort, Bhatta, L. D., Baral, H., Rai, R. K., Dhakal, M., Rucevska, I., & Adhikari, R. (2015). Assessing community values to support mapping of ecosystem services in the Koshi river basin, Nepal. Ecosystem Services, 13, 70–80. https://doi.org/10.1016/j.ecoser.2014.11.004
23. Zomer, Trabucco, A., Metzger, M. J., Wang, M., Oli, K. P., & Xu, J. (2014). Projected climate change impacts on spatial distribution of bioclimatic zones and ecoregions within the Kailash Sacred Landscape of China, India, Nepal. Climatic Change, 125(3-4), 445–460. https://doi.org/10.1007/s10584-014-1176-2
24. Kandel, Tshering, D., Uddin, K., Lhamtshok, T., Aryal, K., Karki, S., Sharma, B., & Chettri, N. (2018). Understanding social–ecological interdependence using ecosystem services perspective in Bhutan, Eastern Himalayas. Ecosphere (Washington, D.C), 9(2), e02121–n/a. https://doi.org/10.1002/ecs2.2121
25. Gupta, Everard, M., & Namchu, C. V. (2021). Declining native fish, diminishing livelihood security: the predicament of Indian Himalayan communities. International Journal of River Basin Management, 19(2), 255–259. https://doi.org/10.1080/15715124.2020.1790578
26. Mukherji, Molden, D., Nepal, S., Rasul, G., & Wagnon, P. (2015). Himalayan waters at the crossroads: issues and challenges. International Journal of Water Resources Development, 31(2), 151–160. https://doi.org/10.1080/07900627.2015.1040871
27. Macchi, Gurung, A. M., & Hoermann, B. (2015). Community perceptions and responses to climate variability and change in the Himalayas. Climate and Development, 7(5), 414–425. https://doi.org/10.1080/17565529.2014.966046
28. Deep Narayan Pandey. (2002). Global climate change and carbon management in multifunctional forests. Current Science (Bangalore), 83(5), 593–602.
29. Bhattacharjee, Anadón, J., Lohman, D., Doleck, T., Lakhankar, T., Shrestha, B., Thapa, P., Devkota, D., Tiwari, S., Jha, A., Siwakoti, M., Devkota, N., Jha, P., & Krakauer, N. (2017). The Impact of Climate Change on Biodiversity in Nepal: Current Knowledge, Lacunae, and Opportunities. Climate (Basel), 5(4), 80–. https://doi.org/10.3390/cli5040080
30. Talukder, Matthew, R., vanLoon, G. W., Bunch, M. J., Hipel, K. W., & Orbinski, J. (2021). Melting of Himalayan glaciers and planetary health. Current Opinion in Environmental Sustainability, 50, 98–108. https://doi.org/10.1016/j.cosust.2021.02.002
31. Xu, Liu, W., Zhao, D., Hao, Y., Xia, A., Yan, N., & Zeng, Y. (2022). Remote Sensing-based Spatiotemporal Distribution of Grassland Aboveground Biomass and Its Response to Climate Change in the Hindu Kush Himalayan Region. Chinese Geographical Science, 32(5), 759–775. https://doi.org/10.1007/s11769-022-1299-8
32. Pariva Dobriyal, Srishti Badola, Syed Ainul Hussain, & Ruchi Badola. (2022). Toward SDGs: Forest, Market and Human Wellbeing Nexus in Indian Western Himalayas. Frontiers in Ecology and Evolution, 10. https://doi.org/10.3389/fevo.2022.846549
33. Tyagi, & Kumar, M. (2022). The resilience of Indian Western Himalayan forests to regime shift: Are they reaching towards no return point? Ecological Informatics, 69, 101644–. https://doi.org/10.1016/j.ecoinf.2022.101644
34. Yadav, Negi, P. S., & Singh, J. (2021). Climate change and plant biodiversity in Himalaya, India. Proceedings of the Indian National Science Academy, 87(2), 234–259. https://doi.org/10.1007/s43538-021-00034-5
35. Chakraborty. (2020). Mountains as a Global Heritage: Arguments for Conserving the Natural Diversity of Mountain Regions. Heritage, 3(2), 198–207. https://doi.org/10.3390/heritage3020012
36. Dubey, Sharma, A., Panchariya, V. K., Goyal, M. K., Surampalli, R. Y., & Zhang, T. C. (2021). Regional sustainable development of renewable natural resources using Net Primary Productivity on a global scale. Ecological Indicators, 127, 107768–. https://doi.org/10.1016/j.ecolind.2021.107768
37. Layek, Narzari, R., Hazarika, S., Das, A., Rangappa, K., Devi, S., Balusamy, A., Saha, S., Mandal, S., Idapuganti, R. G., Babu, S., Choudhury, B. U., & Mishra, V. K. (2022). Prospects of Biochar for Sustainable Agriculture and Carbon Sequestration: An Overview for Eastern Himalayas. Sustainability (Basel, Switzerland), 14(11), 6684–. https://doi.org/10.3390/su14116684
38. Thakur, Negi, V. S., Pathak, R., Dhyani, R., Durgapal, K., & Rawal, R. S. (2020). Indicator based integrated vulnerability assessment of community forests in Indian west Himalaya. Forest Ecology and Management, 457, 117674–. https://doi.org/10.1016/j.foreco.2019.117674
39. Shrestha, Shrestha, B. B., & Vaclavik, T. (2019). Climate change amplifies plant invasion hotspots in Nepal. Diversity & Distributions, 25(10), 1599–1612. https://doi.org/10.1111/ddi.12963
40. Pandey, Kumar, P., Archie, K. M., Gupta, A. K., Joshi, P. K., Valente, D., & Petrosillo, I. (2018). Climate change adaptation in the western-Himalayas: Household level perspectives on impacts and barriers. Ecological Indicators, 84, 27–37. https://doi.org/10.1016/j.ecolind.2017.08.021
41. Negi, Tiwari, D. C., Singh, L., Thakur, S., & Bhatt, I. D. (2021). Review and synthesis of climate change studies in the Himalayan region. Environment, Development and Sustainability, 24(9), 10471–10502. https://doi.org/10.1007/s10668-021-01880-5
42. Singh, & Goyal, M. K. (2017). Curve number modifications and parameterization sensitivity analysis for reducing model uncertainty in simulated and projected streamflows in a Himalayan catchment. Ecological Engineering, 108, 17–29. https://doi.org/10.1016/j.ecoleng.2017.08.002
43. Baig, Khan, A. A., Ali, A., Khan, M. Z., Ahmed, S., Shah, G. M., & Ali, G. (2020). Enhancing socioeconomic resilience and climate adaptation through value chain development of mountain products in Hindu Kush Himalayas. Environment, Development and Sustainability, 23(6), 8451–8473. https://doi.org/10.1007/s10668-020-00975-9
44. Dahal, Shrestha, M. L., Panthi, J., & Pradhananga, D. (2020). Modeling the future impacts of climate change on water availability in the Karnali River Basin of Nepal Himalaya. Environmental Research, 185, 109430–109430. https://doi.org/10.1016/j.envres.2020.109430
45. Paudel, Wang, Z., Zhang, Y., Rai, M. K., & Paul, P. K. (2021). Climate Change and Its Impacts on Farmer’s Livelihood in Different Physiographic Regions of the Trans-Boundary Koshi River Basin, Central Himalayas. International Journal of Environmental Research and Public Health, 18(13), 7142–. https://doi.org/10.3390/ijerph18137142
46. Gu, Zhang, Y., Liu, L., Li, L., Li, S., Zhang, B., Cui, B., & Rai, M. K. (2021). Qualifying Land Use and Land Cover Dynamics and Their Impacts on Ecosystem Service in Central Himalaya Transboundary Landscape Based on Google Earth Engine. Land (Basel), 10(2), 173–. https://doi.org/10.3390/land10020173
47. Chauhan, Uniyal, V. P., Chandra, A., Thakur, P., & Mehrwar, V. (2021). Preliminary assessment and conservation of insect pollinators through community participation in the Lahaul and Spiti district of Himachal Pradesh, India. Current Science (Bangalore), 120(5), 883–887. <https://doi.org/10.18520/cs/v120/i5/883-887>
48. Penjor, U., *et al*. (2022). Effects of land use and climate change on functional and phylogenetic diversity of terrestrial vertebrates in a Himalayan biodiversity hotspot. *Diversity and Distributions.*
49. Choden, Nitschke, C. R., Stewart, S. B., & Keenan, R. J. (2021). The potential impacts of climate change on the distribution of key tree species and Cordyceps in Bhutan: Implications for ecological functions and rural livelihoods. Ecological Modelling, 455, 109650–. https://doi.org/10.1016/j.ecolmodel.2021.109650
50. Everard, Gupta, N., Chapagain, P. S., Shrestha, B. B., Preston, G., & Tiwari, P. (2018). Can control of invasive vegetation improve water and rural livelihood security in Nepal? Ecosystem Services, 32, 125–133. https://doi.org/10.1016/j.ecoser.2018.07.004
51. Thapa, Baral, S., Hu, Y., Huang, Z., Yue, Y., Dhakal, M., Jnawali, S. R., Chettri, N., Racey, P. A., Yu, W., & Wu, Y. (2021). Will climate change impact distribution of bats in Nepal Himalayas? A case study of five species. Global Ecology and Conservation, 26, e01483–. <https://doi.org/10.1016/j.gecco.2021.e01483>
52. Das, BC. (2012). Ecosystem services and conservation value of Himalayan insects with special reference to Uttarakhand under the influence of the changing climate. [4th Symposium of Biodiversity and Natural Heritage of the Himalaya](https://www.webofscience.com/wos/woscc/general-summary?queryJson=%5B%7B%22rowBoolean%22:null,%22rowField%22:%22CF%22,%22rowText%22:%224th%20Symposium%20of%20Biodiversity%20and%20Natural%20Heritage%20of%20the%20Himalaya%22%7D%5D). 4, 121-127
53. Dhyani, Kadaverugu, R., & Pujari, P. (2020). Predicting impacts of climate variability on Banj oak (Quercus leucotrichophora A. Camus) forests: understanding future implications for Central Himalayas. Regional Environmental Change, 20(4). https://doi.org/10.1007/s10113-020-01696-5
54. Rawat, Maikhuri, R. K., Bahuguna, Y. M., Jugran, A. K., Maletha, A., Jha, N. K., Phondani, P. C., Dhyani, D., Pharswan, D. S., & Chamoli, S. (2022). Rejuvenating ecosystem services through reclaiming degraded land for sustainable societal development: Implications for conservation and human wellbeing. Land Use Policy, 112, 105804–. https://doi.org/10.1016/j.landusepol.2021.105804
55. Bagchi, & Ritchie, M. E. (2010). Introduced grazers can restrict potential soil carbon sequestration through impacts on plant community composition. Ecology Letters, 13(8), 959–968. https://doi.org/10.1111/j.1461-0248.2010.01486.x
56. Verma, Schmidt-Vogt, D., De Alban, J. D. T., Lim, C. L., & Webb, E. L. (2021). Drivers and mechanisms of forest change in the Himalayas. Global Environmental Change, 68, 102244–. https://doi.org/10.1016/j.gloenvcha.2021.102244
57. Kamaljit S. Bawa, & Reinmar Seidler. (2015). DEFORESTATION AND SUSTAINABLE MIXED-USE LANDSCAPES: A VIEW FROM THE EASTERN HIMALAYA. Annals of the Missouri Botanical Garden, 100(3), 141–149.
58. Paudyal, Baral, H., Lowell, K., & Keenan, R. J. (2017). Ecosystem services from community-based forestry in Nepal: Realising local and global benefits. Land Use Policy, 63, 342–355. https://doi.org/10.1016/j.landusepol.2017.01.046
59. Cui, Zhang, Y., Wang, Z., Gu, C., Liu, L., Wei, B., Gong, D., & Rai, M. K. (2022). Ecological Risk Assessment of Transboundary Region Based on Land-Cover Change: A Case Study of Gandaki River Basin, Himalayas. Land (Basel), 11(5), 638–. https://doi.org/10.3390/land11050638
60. Negi. (2022). Trees, forests and people: The Central Himalayan case of forest ecosystem services. Trees, Forests and People (Online), 8, 100222–. <https://doi.org/10.1016/j.tfp.2022.100222>
61. Singh, SP., Sharma, CM. (2009). Tropical ecology: An overview. *Tropical Ecology*. 50(1), 7-21.
62. Khan, Page, S. E., Ahmad, H., & Harper, D. M. (2013). Sustainable utilization and conservation of plant biodiversity in montane ecosystems: the western Himalayas as a case study. Annals of Botany, 112(3), 479–501. https://doi.org/10.1093/aob/mct125
63. Romshoo, Murtaza, K. O., Shah, W., Ramzan, T., Ameen, U., & Bhat, M. H. (2022). Anthropogenic climate change drives melting of glaciers in the Himalaya. Environmental Science and Pollution Research International, 29(35), 52732–52751. https://doi.org/10.1007/s11356-022-19524-0
64. Stratford, Acreman, M. C., & Rees, H. G. (2011). simple method for assessing the vulnerability of wetland ecosystem services. Hydrological Sciences Journal, 56(8), 1485–1500. https://doi.org/10.1080/02626667.2011.630669
65. Rawat, Arunachalam, K., Arunachalam, A., Alatalo, J. M., Kumar, U., Simon, B., Hufnagel, L., Micheli, E., & Pandey, R. (2020). Relative contribution of plant traits and soil properties to the functioning of a temperate forest ecosystem in the Indian Himalayas. Catena (Giessen), 194, 104671–. https://doi.org/10.1016/j.catena.2020.104671
66. Everard, Kataria, G., Kumar, S., & Gupta, N. (2020). Assessing livelihood-ecosystem interdependencies and natural resource governance in a tribally controlled region of India’s north-eastern middle Himalayas. Environment, Development and Sustainability, 23(5), 7772–7790. https://doi.org/10.1007/s10668-020-00945-1
67. Chakraborty. (2019). Mountains as vulnerable places: a global synthesis of changing mountain systems in the Anthropocene. GeoJournal, 86(2), 585–604. https://doi.org/10.1007/s10708-019-10079-1
68. Dorji, Brookes, J., Facelli, J., Sears, R., Norbu, T., Dorji, K., Chhetri, Y., & Baral, H. (2019). Socio-Cultural Values of Ecosystem Services from Oak Forests in the Eastern Himalaya. Sustainability (Basel, Switzerland), 11(8), 2250–. https://doi.org/10.3390/su11082250
69. Kotru, Shakya, B., Joshi, S., Gurung, J., Ali, G., Amatya, S., & Pant, B. (2020). Biodiversity Conservation and Management in the Hindu Kush Himalayan Region: Are Transboundary Landscapes a Promising Solution? Mountain Research and Development, 40(2), A15–A23. https://doi.org/10.1659/MRD-JOURNAL-D-19-00053.1
70. Shukla, Sachdeva, K., & Joshi, P. K. (2017). Demystifying vulnerability assessment of agriculture communities in the Himalayas: a systematic review. Natural Hazards (Dordrecht), 91(1), 409–429. https://doi.org/10.1007/s11069-017-3120-z
71. Khandekar, Gorti, G., Bhadwal, S., & Rijhwani, V. (2019). Perceptions of climate shocks and gender vulnerabilities in the Upper Ganga Basin. Environmental Development, 31, 97–109. <https://doi.org/10.1016/j.envdev.2019.02.001>
72. Exler, N., *et al.* Biodiversity and Remote Sensing in Relation to Diverging Scales in the BRAHMATWINN Project. 3rd International Symposium on Biodiversity and Natural Heritage of the Himalaya.
73. Ncube, Visser, A., & Beevers, L. (2018). A Framework for Assessing Instream Supporting Ecosystem Services Based on Hydroecological Modelling. Water (Basel), 10(9), 1247–. https://doi.org/10.3390/w10091247
74. Chug, Pathak, A., Indu, J., Jain, S. K., Jain, S. K., Dimri, A. P., Niyogi, D., & Ghosh, S. (2020). Observed Evidence for Steep Rise in the Extreme Flow of Western Himalayan Rivers. Geophysical Research Letters, 47(15). https://doi.org/10.1029/2020GL087815
75. Miller, Rana, P., & Benson Wahlén, C. (2017). A Crystal Ball for Forests?: Analyzing the Social-Ecological Impacts of Forest Conservation and Management over the Long Term. Environment and Society, 8(1), 40–62. https://doi.org/10.3167/ares.2017.080103
76. Mukherji, Sinisalo, A., Nüsser, M., Garrard, R., & Eriksson, M. (2019). Contributions of the cryosphere to mountain communities in the Hindu Kush Himalaya: a review. Regional Environmental Change, 19(5), 1311–1326. https://doi.org/10.1007/s10113-019-01484-w
77. Rasul, Hussain, A., Adhikari, L., & Molden, D. J. (2022). Conserving agrobiodiversity for sustainable food systems in the Hindu Kush Himalaya. International Journal of Agricultural Sustainability, ahead-of-print(ahead-of-print), 1–19. https://doi.org/10.1080/14735903.2022.2057642
78. Negi, Singh, P., & Singh, S. P. (2021). Atmospheric Warming-Associated Phenological Earliness Does Not Increase the Length of Growing Season in Himalayan Trees. Forest Science, 67(6), 694–700. https://doi.org/10.1093/forsci/fxab040
79. Reang, Hazarika, A., Sileshi, G. W., Pandey, R., Das, A. K., & Nath, A. J. (2021). Assessing tree diversity and carbon storage during land use transitioning from shifting cultivation to indigenous agroforestry systems: Implications for REDD plus initiatives. Journal of Environmental Management, 298. https://doi.org/10.1016/j.jenvman.2021.113470
80. Ishfaq Ahmad Wani, Sajid Khan, Susheel Verma, Fahad A. Al-Misned, Hesham M. Shafik, & Hamed A. El-Serehy. (2022). Predicting habitat suitability and niche dynamics of Dactylorhiza hatagirea and Rheum webbianum in the Himalaya under projected climate change. Scientific Reports, 12(1), 1–18. https://doi.org/10.1038/s41598-022-16837-5
81. Rasul. (2014). Food, water, and energy security in South Asia: A nexus perspective from the Hindu Kush Himalayan region. Environmental Science & Policy, 39, 35–48. https://doi.org/10.1016/j.envsci.2014.01.010
82. Naudiyal, & Schmerbeck, J. (2021). Potential distribution of oak forests in the central Himalayas and implications for future ecosystem services supply to rural communities. Ecosystem Services, 50, 101310–. https://doi.org/10.1016/j.ecoser.2021.101310
83. Deep Narayan Pandey. (2007). Multifunctional agroforestry systems in India. Current Science (Bangalore), 92(4), 455–463.
84. Sharma, G., *et al*. Comparative analysis on the socio-ecological and economic potentials of traditional agroforestry systems in the Sikkim Himalaya. Tropical Ecology. 57(4), 751-764.
85. Regmi, Bhusal, J., Gurung, P., Zulkafli, Z., Karpouzoglou, T., Ochoa Tocachi, B., Buytaert, W., & Mao, F. (2019). Learning to cope with water variability through participatory monitoring: the case study of the Mountainous region, Nepal. https://doi.org/10.26491/mhwm/106021
86. Shrestha, Bajracharya, S. R., Sharma, A. R., Duo, C., & Kulkarni, A. (2017). Observed trends and changes in daily temperature and precipitation extremes over the Koshi river basin 1975–2010. International Journal of Climatology, 37(2), 1066–1083. https://doi.org/10.1002/joc.4761
87. Karimi, Nawaz, M. A., Naseem, S., Akrem, A., Ali, H., Dangles, O., & Ali, Z. (2021). The response of culturally important plants to experimental warming and clipping in Pakistan Himalayas. PloS One, 16(5), e0237893–e0237893. https://doi.org/10.1371/journal.pone.0237893
88. Malik, & Rai, S. C. (2019). Drivers of land use/cover change and its impact on Pong Dam wetland. Environmental Monitoring and Assessment, 191(4), 203–214. https://doi.org/10.1007/s10661-019-7347-x
89. Bhat, & Pandit, A. K. (2021). Water quality assessment and monitoring of Kashmir Himalayan freshwater springs-A case study. Aquatic Ecosystem Health & Management, 23(3), 274–287. https://doi.org/10.1080/14634988.2020.1816771
90. Rangwala, Sinsky, E., & Miller, J. R. (2016). Variability in projected elevation dependent warming in boreal midlatitude winter in CMIP5 climate models and its potential drivers. Climate Dynamics, 46(7-8), 2115–2122. https://doi.org/10.1007/s00382-015-2692-0
91. Sharma, Chettri, N., & Oli, K. P. (2010). Mountain biodiversity conservation and management: a paradigm shift in policies and practices in the Hindu Kush-Himalayas. Ecological Research, 25(5), 909–923. <https://doi.org/10.1007/s11284-010-0747-6>
92. Dhyani, S., *et al.* (2020). Impact of anthropogenic interferences on species composition, regeneration and stand quality in moist temperate forests of Central Himalaya. Tropical Ecology. 60(4), 539-551
93. Pradhan, Manohar K, A., Vineeta, Sarkar, B. C., Bhat, J. A., Shukla, G., & Chakravarty, S. (2020). Ecosystem services of urban green sites- A case study from Eastern Himalayan foothills. Trees, Forests and People (Online), 2, 100029–. <https://doi.org/10.1016/j.tfp.2020.100029>
94. Gull, S., et al. (2022). Assessing land use/land cover change detection of north-eastern watersheds of Kashmir valley using GIS and remote sensing techniques. Water Practice and Technoclogy. 17(8), 1603-1614
95. Wahid, Shrestha, A. B., Murthy, M. S. R., Matin, M., Zhang, J., & Siddiqui, O. (2014). Regional Water Security in the Hindu Kush Himalayan Region: Role of Geospatial Science and Tools. International Archives of the Photogrammetry, Remote Sensing and Spatial Information Sciences., XL-8(8), 1331–1340. https://doi.org/10.5194/isprsarchives-XL-8-1331-2014
96. Ingty. (2021). Pastoralism in the highest peaks: Role of the traditional grazing systems in maintaining biodiversity and ecosystem function in the alpine Himalaya. PloS One, 16(1), e0245221–e0245221. https://doi.org/10.1371/journal.pone.0245221
97. Suresh K. Rana, Ranbeer S. Rawal, Bhawana Dangwal, Indra D. Bhatt, & Trevor D. Price. (2021). 200 Years of Research on Himalayan Biodiversity: Trends, Gaps, and Policy Implications. Frontiers in Ecology and Evolution, 8. https://doi.org/10.3389/fevo.2020.603422
98. Chakraborty, Joshi, P. K., & Sachdeva, K. (2017). Capturing forest dependency in the central Himalayan region: Variations between Oak (Quercus spp.) and Pine (Pinus spp.) dominated forest landscapes. Ambio, 47(4), 504–522. https://doi.org/10.1007/s13280-017-0947-1
99. Yadav, Gupta, B., Bhutia, P. L., Bisht, J. K., Pattanayak, A., Meena, V. S., Choudhary, M., & Tiwari, P. (2019). Biomass and carbon budgeting of sustainable agroforestry systems as ecosystem service in Indian Himalayas. International Journal of Sustainable Development and World Ecology, 26(5), 460–470. https://doi.org/10.1080/13504509.2019.1600597
100. Chakraborty, & Ghosal, S. (2022). Status of mountain-tourism and research in the Indian Himalayan Region: a systematic review. Asia-Pacific Journal of Regional Science. https://doi.org/10.1007/s41685-022-00243-w
101. Buechler, Sen, D., Khandekar, N., & Scott, C. (2016). Re-Linking Governance of Energy with Livelihoods and Irrigation in Uttarakhand, India. Water (Basel), 8(10), 437–437. https://doi.org/10.3390/w8100437
102. O’Neill, Chhetri, P. K., Chhetri, B., & Rana, S. K. (2020). Establishing ecological baselines around a temperate Himalayan peatland. Wetlands Ecology and Management, 28(2), 375–388. https://doi.org/10.1007/s11273-020-09710-7
103. Zaman, Iqbal, A., Shaukat, A., Nazir, R., Pervez, A., Bilal, M., Faridullah, Rizwan, M., Ali, S., Alkahtani, S., Abdel-Daim, M. M., & Hafeez, F. (2021). Assessing the N Cycling Ecosystem Function-Processes and the Involved Functional Guilds upon Plant Litter Amendment in Lower Himalaya. Polish Journal of Environmental Studies, 30(1), 917–926. https://doi.org/10.15244/pjoes/122771
104. Ghosh. (2021). A framework to assess the benefits and challenges of ecosystem services of a reservoir-based wetland in the Himalayan foothills. Environmental Development, 40, 100669–. https://doi.org/10.1016/j.envdev.2021.100669
105. GUPTA, GRAINGER, M., DUNN, J. C., SANDERSON, R., & MCGOWAN, P. J. K. (2022). Conservation of Galliformes in the Greater Himalaya: is there a need for a higher-quality evidence-base? Bird Conservation International, 32(3), 360–369. https://doi.org/10.1017/S0959270921000514
106. Kumar, Kumar, P., Singh, H., & Kumar, N. (2021). Modulation of plant functional traits under essential plant nutrients during seasonal regime in natural forests of Garhwal Himalayas. Plant and Soil, 465(1-2), 197–212. https://doi.org/10.1007/s11104-021-05003-x
107. Tiwari, & Joshi, B. (2012). Natural and socio-economic factors affecting food security in the Himalayas. Food Security, 4(2), 195–207. https://doi.org/10.1007/s12571-012-0178-z
108. Wani, Verma, S., Gupta, R., Ganaie, M. M., Nigam, G., Shafik, H. M., & Al-Misned, F. A. (2022). Nutrient Analysis and Species Diversity of Alpine Grasslands: A Comparative Analysis of Less Studied Biodiversity Hotspots. Sustainability (Basel, Switzerland), 14(2), 887–. https://doi.org/10.3390/su14020887
109. Chaudhary, Wang, Y., Dixit, A. M., Khanal, N. R., Xu, P., Yan, K., Liu, Q., Lu, Y., & Li, M. (2019). Eco-Environmental Risk Evaluation for Land Use Planning in Areas of Potential Farmland Abandonment in the High Mountains of Nepal Himalayas. Sustainability (Basel, Switzerland), 11(24), 6931–. https://doi.org/10.3390/su11246931
110. Sharma, Areendran, G., Raj, K., Sharma, A., & Joshi, P. K. (2016). Multitemporal analysis of forest fragmentation in Hindu Kush Himalaya—a case study from Khangchendzonga Biosphere Reserve, Sikkim, India. Environmental Monitoring and Assessment, 188(10), 596–596. https://doi.org/10.1007/s10661-016-5577-8
111. Mohanty, & Tare, V. (2022). Anthropogenic Interventions in Watersheds on River Flow Health: Assessment Using Bootstrapped Principal Component Analysis. Journal of Water Resources Planning and Management, 148(1). https://doi.org/10.1061/(ASCE)WR.1943-5452.0001499
112. Meraj, Farooq, M., Singh, S. K., Islam, M. N., & Kanga, S. (2021). Modeling the sediment retention and ecosystem provisioning services in the Kashmir valley, India, Western Himalayas. Modeling Earth Systems and Environment, 8(3), 3859–3884. https://doi.org/10.1007/s40808-021-01333-y
113. Murthy, Bajracharya, B., Pradhan, S., Shestra, B., Bajracharya, R., Shakya, K., Wesselmann, S., Ali, M., & Bajracharya, S. (2014). Adoption of Geospatial Systems towards evolving Sustainable Himalayan Mountain Development. International Archives of the Photogrammetry, Remote Sensing and Spatial Information Sciences., XL-8(8), 1319–1324. https://doi.org/10.5194/isprsarchives-XL-8-1319-2014
114. Vercruysse, & Grabowski, R. C. (2021). Human impact on river planform within the context of multi-timescale river channel dynamics in a Himalayan river system. Geomorphology (Amsterdam, Netherlands), 381, 107659–. https://doi.org/10.1016/j.geomorph.2021.107659
115. Penjor, Jamtsho, R., & Sherub, S. (2022). Anthropogenic land‐use change shapes bird diversity along the eastern Himalayan altitudinal gradient. The Journal of Applied Ecology, 59(3), 847–859. https://doi.org/10.1111/1365-2664.14101
116. Sharma, Acharya, B. K., Sharma, G., Valente, D., Pasimeni, M. R., Petrosillo, I., & Selvan, T. (2020). Land use effect on butterfly alpha and beta diversity in the Eastern Himalaya, India. Ecological Indicators, 110, 105605–. https://doi.org/10.1016/j.ecolind.2019.105605
117. Hanspach, Jamila Haider, L., Oteros‐Rozas, E., Stahl Olafsson, A., Gulsrud, N. M., Raymond, C. M., Torralba, M., Martín‐López, B., Bieling, C., García‐Martín, M., Albert, C., Beery, T. H., Fagerholm, N., Díaz‐Reviriego, I., Drews‐Shambroom, A., Plieninger, T., & Rozzi, R. (2020). Biocultural approaches to sustainability: A systematic review of the scientific literature. People and Nature (Hoboken, N.J.), 2(3), 643–659. https://doi.org/10.1002/pan3.10120
118. Parajuli, O’Brien, M. J., Timilsina, B., Pugnaire, F. I., Schöb, C., & Ghimire, S. K. (2021). Facilitation by a dwarf shrub enhances plant diversity of human-valued species at high elevations in the Himalayas of Nepal. Basic and Applied Ecology, 54, 23–36. https://doi.org/10.1016/j.baae.2021.04.004
119. Pandey, Singh, G., Palni, S., Chandra, N., Rawat, J., & Singh, A. P. (2021). Application of remote sensing in alpine grasslands cover mapping of western Himalaya, Uttarakhand, India. Environmental Monitoring and Assessment, 193(4), 166–166. https://doi.org/10.1007/s10661-021-08956-9
120. Mishra, & Chaudhuri, G. (2015). Spatio-temporal analysis of trends in seasonal vegetation productivity across Uttarakhand, Indian Himalayas, 2000–2014. Applied Geography (Sevenoaks), 56, 29–41. https://doi.org/10.1016/j.apgeog.2014.10.007
121. Bajracharya, Murthy, M. S. R., & Shrestha, B. (2014). SERVIR HIMALYA: Enabling Improved Environmental Management and Livelihoods in the HKH. International Archives of the Photogrammetry, Remote Sensing and Spatial Information Sciences., XL-8(8), 1277–1281. https://doi.org/10.5194/isprsarchives-XL-8-1277-2014
122. Pandey, Kumar De, H., Dubey, S. K., Kumar, B., Dobhal, S., & Adhiguru, P. (2020). Indigenous people’s attachment to shifting cultivation in the Eastern Himalayas, India: A cross-sectional evidence. Forest Policy and Economics, 111, 102046–. https://doi.org/10.1016/j.forpol.2019.102046
123. Tiwari, Uprety, Y., & Rana, S. K. (2019). Plant endemism in the Nepal Himalayas and phytogeographical implications. Plant Diversity, 41(3), 174–182. https://doi.org/10.1016/j.pld.2019.04.004
124. Rawat, Khanduri, V. P., Singh, B., Riyal, M. K., Thakur, T. K., Kumar, M., & Cabral-Pinto, M. M. (2022). Variation in carbon stock and soil properties in different Quercus leucotrichophora forests of Garhwal Himalaya. Catena (Giessen), 213, 106210–. https://doi.org/10.1016/j.catena.2022.106210
125. Roberts, Cooper, W. J., & Luther, D. (2021). Global assessment of forest quality for threatened terrestrial vertebrate species in need of conservation translocation programs. PloS One, 16(4), e0249378–e0249378. https://doi.org/10.1371/journal.pone.0249378
126. Zhang, Jiang, F., Li, G., Qin, W., Li, S., Gao, H., Cai, Z., Lin, G., & Zhang, T. (2019). Maxent modeling for predicting the spatial distribution of three raptors in the Sanjiangyuan National Park, China. Ecology and Evolution, 9(11), 6643–6654. https://doi.org/10.1002/ece3.5243
127. Jewell Lund, Richard R. Forster, Summer B. Rupper, Elias J. Deeb, H. P. Marshall, Muhammad Zia Hashmi, & Evan Burgess. (2020). Mapping Snowmelt Progression in the Upper Indus Basin With Synthetic Aperture Radar. Frontiers in Earth Science (Lausanne), 7. https://doi.org/10.3389/feart.2019.00318
128. Kumar, Bijalwan, A., Singh, B., Rawat, D., Yewale, A. G., Riyal, M. K., & Thakur, T. K. (2021). Comparison of Carbon Sequestration Potential of Quercus leucotrichophora–Based Agroforestry Systems and Natural Forest in Central Himalaya, India. Water, Air, and Soil Pollution, 232(9). <https://doi.org/10.1007/s11270-021-05294-x>
129. Chaudhard, S., et al. (2022). Protected areas in the Hindu Kush Himalaya: A regional assessment of the status, distribution, and gaps. Conservation Science and Practice.
130. Qazi. (2020). Hydrological functioning of forested catchments, Central Himalayan Region, India. Forest Ecosystems, 7(1). https://doi.org/10.1186/s40663-020-00275-8
131. Khanday, Bhat, S. U., Islam, S. T., & Sabha, I. (2021). Identifying lithogenic and anthropogenic factors responsible for spatio-seasonal patterns and quality evaluation of snow melt waters of the River Jhelum Basin in Kashmir Himalaya. Catena (Giessen), 196, 104853–. https://doi.org/10.1016/j.catena.2020.104853

Agriculture AND Himalaya* AND Ecosystem service*

1. Kamaljit S. Bawa, & Reinmar Seidler. (2015). DEFORESTATION AND SUSTAINABLE MIXED-USE LANDSCAPES: A VIEW FROM THE EASTERN HIMALAYA. Annals of the Missouri Botanical Garden, 100(3), 141–149.
2. Kandel, Tshering, D., Uddin, K., Lhamtshok, T., Aryal, K., Karki, S., Sharma, B., & Chettri, N. (2018). Understanding social–ecological interdependence using ecosystem services perspective in Bhutan, Eastern Himalayas. Ecosphere (Washington, D.C), 9(2), e02121–n/a. https://doi.org/10.1002/ecs2.2121
3. Layek, Narzari, R., Hazarika, S., Das, A., Rangappa, K., Devi, S., Balusamy, A., Saha, S., Mandal, S., Idapuganti, R. G., Babu, S., Choudhury, B. U., & Mishra, V. K. (2022). Prospects of Biochar for Sustainable Agriculture and Carbon Sequestration: An Overview for Eastern Himalayas. Sustainability (Basel, Switzerland), 14(11), 6684–. https://doi.org/10.3390/su14116684
4. Everard, Gupta, N., Scott, C. A., Tiwari, P. C., Joshi, B., Kataria, G., & Kumar, S. (2018). Assessing livelihood-ecosystem interdependencies and natural resource governance in Indian villages in the Middle Himalayas. Regional Environmental Change, 19(1), 165–177. https://doi.org/10.1007/s10113-018-1391-x
5. Shrestha, Ye, Q., & Khadka, N. (2019). Assessment of Ecosystem Services Value Based on Land Use and Land Cover Changes in the Transboundary Karnali River Basin, Central Himalayas. Sustainability (Basel, Switzerland), 11(11), 3183–. https://doi.org/10.3390/su11113183
6. Regmi, Bhusal, J., Gurung, P., Zulkafli, Z., Karpouzoglou, T., Ochoa Tocachi, B., Buytaert, W., & Mao, F. (2019). Learning to cope with water variability through participatory monitoring: the case study of the Mountainous region, Nepal. https://doi.org/10.26491/mhwm/106021
7. Aukema, Pricope, N. G., Husak, G. J., & Lopez-Carr, D. (2017). Biodiversity Areas under Threat: Overlap of Climate Change and Population Pressures on the World’s Biodiversity Priorities. PloS One, 12(1), e0170615–e0170615. https://doi.org/10.1371/journal.pone.0170615
8. Shukla, Sachdeva, K., & Joshi, P. K. (2017). Demystifying vulnerability assessment of agriculture communities in the Himalayas: a systematic review. Natural Hazards (Dordrecht), 91(1), 409–429. https://doi.org/10.1007/s11069-017-3120-z
9. Rasul, Hussain, A., Adhikari, L., & Molden, D. J. (2022). Conserving agrobiodiversity for sustainable food systems in the Hindu Kush Himalaya. International Journal of Agricultural Sustainability, ahead-of-print(ahead-of-print), 1–19. https://doi.org/10.1080/14735903.2022.2057642
10. Birch, Thapa, I., Balmford, A., Bradbury, R. B., Brown, C., Butchart, S. H. M., Gurung, H., Hughes, F. M. R., Mulligan, M., Pandeya, B., Peh, K. S.-H., Stattersfield, A. J., Walpole, M., & Thomas, D. H. L. (2014). What benefits do community forests provide, and to whom? A rapid assessment of ecosystem services from a Himalayan forest, Nepal. Ecosystem Services, 8, 118–127. https://doi.org/10.1016/j.ecoser.2014.03.005
11. Chettri, Sharma, E., Shakya, B., & Bajracharya, B. (2007). Developing Forested Conservation Corridors in the Kangchenjunga Landscape, Eastern Himalaya. Mountain Research and Development, 27(3), 211–214. https://doi.org/10.1659/mrd.0923
12. Gurung, Chettri, N., Sharma, E., Ning, W., Chaudhary, R. P., Badola, H. K., Wangchuk, S., Uprety, Y., Gaira, K. S., Bidha, N., Phuntsho, K., Uddin, K., & Shah, G. M. (2019). Evolution of a transboundary landscape approach in the Hindu Kush Himalaya: Key learnings from the Kangchenjunga Landscape. Global Ecology and Conservation, 17, e00599–. https://doi.org/10.1016/j.gecco.2019.e00599
13. Reang, Hazarika, A., Sileshi, G. W., Pandey, R., Das, A. K., & Nath, A. J. (2021). Assessing tree diversity and carbon storage during land use transitioning from shifting cultivation to indigenous agroforestry systems: Implications for REDD plus initiatives. Journal of Environmental Management, 298. https://doi.org/10.1016/j.jenvman.2021.113470
14. Huber, Posch, E., Bell, R., Hoferl, K. M., Steiger, R., Stotten, R., Tasser, E., & Leitinger, G. (2021). Two perspectives - one goal: resilience research in protected mountain regions. Eco.mont, 13(2), 12–20. https://doi.org/10.1553/eco.mont-13-2s12
15. Malik, & Rai, S. C. (2019). Drivers of land use/cover change and its impact on Pong Dam wetland. Environmental Monitoring and Assessment, 191(4), 203–214. https://doi.org/10.1007/s10661-019-7347-x
16. Penjor, Jamtsho, R., & Sherub, S. (2022). Anthropogenic land‐use change shapes bird diversity along the eastern Himalayan altitudinal gradient. The Journal of Applied Ecology, 59(3), 847–859. https://doi.org/10.1111/1365-2664.14101
17. Shrestha, Zhang, L., Sharma, S., Shrestha, S., & Khadka, N. (2022). Effects on ecosystem services value due to land use and land cover change (1990–2020) in the transboundary Karnali River Basin, Central Himalayas. SN Applied Sciences, 4(5), 1–12. https://doi.org/10.1007/s42452-022-05022-y
18. Negi, Maikhuri, R. K., Pharswan, D., Thakur, S., & Dhyani, P. P. (2017). Climate change impact in the Western Himalaya: people’s perception and adaptive strategies. Journal of Mountain Science, 14(2), 403–416. https://doi.org/10.1007/s11629-015-3814-1
19. Qasim, Hubacek, K., & Termansen, M. (2013). Underlying and proximate driving causes of land use change in district Swat, Pakistan. Land Use Policy, 34, 146–157. <https://doi.org/10.1016/j.landusepol.2013.02.008>
20. Penjor, U., et al. (2022). Effects of land use and climate change on functional and phylogenetic diversity of terrestrial vertebrates in a Himalayan biodiversity hotspot. Diversity and Distributions.
21. Kumar Pandey, Dobhal, S., Kumar De, H., Adhiguru, P., Vimla Devi, S., & Mehra, T. S. (2022). Agrobiodiversity in changing shifting cultivation landscapes of the Indian Himalayas: An empirical assessment. Landscape and Urban Planning, 220, 104333–. https://doi.org/10.1016/j.landurbplan.2021.104333
22. Shooshtari, Shayesteh, K., Gholamalifard, M., Azari, M., & López-Moreno, J. I. (2018). Land cover change modelling in Hyrcanian forests, Northern Iran: a landscape pattern and transformation analysis perspective. Cuadernos de Investigación Geográfica, 44(2), 743–761. https://doi.org/10.18172/cig.3279
23. Babu, Singh, R., Avasthe, R. K., Yadav, G. S., Mohapatra, K. P., Selvan, T., Das, A., Singh, V. K., Valente, D., & Petrosillo, I. (2020). Soil carbon dynamics in Indian Himalayan intensified organic rice-based cropping sequences. Ecological Indicators, 114, 106292–. https://doi.org/10.1016/j.ecolind.2020.106292
24. Hayat, Zha, T., Nizam, S. M., Gulzar, S., Khan, A., Iqbal, S., & Khan, M. S. (2020). PRODUCTIVE ROLE OF AGROFORESTRY SYSTEM IN CONTEXT OF ECOSYSTEM SERVICES IN DISTRICT DIR LOWER, PAKISTAN. Pakistan Journal of Botany, 52(4), 1411–1419. https://doi.org/10.30848/PJB2020-4(21)
25. Mukherji, Sinisalo, A., Nüsser, M., Garrard, R., & Eriksson, M. (2019). Contributions of the cryosphere to mountain communities in the Hindu Kush Himalaya: a review. Regional Environmental Change, 19(5), 1311–1326. https://doi.org/10.1007/s10113-019-01484-w
26. Bicudo da Silva, Millington, J. D. A., Moran, E. F., Batistella, M., & Liu, J. (2020). Three decades of land-use and land-cover change in mountain regions of the Brazilian Atlantic Forest. Landscape and Urban Planning, 204, 103948–. https://doi.org/10.1016/j.landurbplan.2020.103948
27. GUPTA, GRAINGER, M., DUNN, J. C., SANDERSON, R., & MCGOWAN, P. J. K. (2022). Conservation of Galliformes in the Greater Himalaya: is there a need for a higher-quality evidence-base? Bird Conservation International, 32(3), 360–369. https://doi.org/10.1017/S0959270921000514
28. Verma, Schmidt-Vogt, D., De Alban, J. D. T., Lim, C. L., & Webb, E. L. (2021). Drivers and mechanisms of forest change in the Himalayas. Global Environmental Change, 68, 102244–. https://doi.org/10.1016/j.gloenvcha.2021.102244
29. Rath, & Ormsby, A. A. (2020). Conservation through Traditional Knowledge: a Review of Research on the Sacred Groves of Odisha, India. Human Ecology, 48(4), 455–463. https://doi.org/10.1007/s10745-020-00173-1
30. Mishra, Rai, A., & Rai, S. C. (2020). Land use and land cover change detection using geospatial techniques in the Sikkim Himalaya, India. The Egyptian Journal of Remote Sensing and Space Sciences, 23(2), 133–143. https://doi.org/10.1016/j.ejrs.2019.02.001
31. Tiwari, & Joshi, B. (2012). Natural and socio-economic factors affecting food security in the Himalayas. Food Security, 4(2), 195–207. https://doi.org/10.1007/s12571-012-0178-z
32. Jana, Dasgupta, S., & Todaria, N. P. (2021). Throughfall and stemflow nutrient flux in deodar and oak forests, Garhwal Himalaya, India. Water Science & Technology. Water Supply, 21(4), 1649–1656. https://doi.org/10.2166/ws.2021.009
33. Dorji, Odeh, I. O. A., & Field, D. J. (2015). Elucidating the complex interrelationships of soil organic carbon fractions with land use/land cover types and landform attributes in a montane ecosystem. Journal of Soils and Sediments, 15(5), 1039–1054. https://doi.org/10.1007/s11368-015-1088-4
34. Yadav, Babu, S., Das, A., Mohapatra, K. P., Singh, R., Avasthe, R. K., & Roy, S. (2020). No-till and mulching enhance energy use efficiency and reduce carbon footprint of a direct-seeded upland rice production system. Journal of Cleaner Production, 271, 122700–. <https://doi.org/10.1016/j.jclepro.2020.122700>
35. Biomass, carbon stock under different production systems in the mid hills of Indian Himalaya
36. Assessing land use/land cover change detection of north-eastern watersheds of Kashmir valley using GIS and remote sensing techniques
37. Bajracharya, Murthy, M. S. R., & Shrestha, B. (2014). SERVIR HIMALYA: Enabling Improved Environmental Management and Livelihoods in the HKH. International Archives of the Photogrammetry, Remote Sensing and Spatial Information Sciences., XL-8(8), 1277–1281. https://doi.org/10.5194/isprsarchives-XL-8-1277-2014
38. Pant, Negi, G. C. S., & Kumar, P. (2017). Macrofauna contributes to organic matter decomposition and soil quality in Himalayan agroecosystems, India. Applied Soil Ecology : a Section of Agriculture, Ecosystems & Environment, 120, 20–29. https://doi.org/10.1016/j.apsoil.2017.07.019
39. Acharya. (2006). Linking Trees on Farms with Biodiversity Conservation in Subsistence Farming Systems in Nepal. Biodiversity and Conservation, 15(2), 631–646. https://doi.org/10.1007/s10531-005-2091-7
40. Suresh K. Rana, Ranbeer S. Rawal, Bhawana Dangwal, Indra D. Bhatt, & Trevor D. Price. (2021). 200 Years of Research on Himalayan Biodiversity: Trends, Gaps, and Policy Implications. Frontiers in Ecology and Evolution, 8. https://doi.org/10.3389/fevo.2020.603422
41. Semwal, Nautiyal, S., Sen, K. ., Rana, U., Maikhuri, R. ., Rao, K. ., & Saxena, K. . (2004). Patterns and ecological implications of agricultural land-use changes: a case study from central Himalaya, India. Agriculture, Ecosystems & Environment, 102(1), 81–92. https://doi.org/10.1016/S0167-8809(03)00228-7
42. Yadav, Gupta, B., Bhutia, P. L., Bisht, J. K., Pattanayak, A., Meena, V. S., Choudhary, M., & Tiwari, P. (2019). Biomass and carbon budgeting of sustainable agroforestry systems as ecosystem service in Indian Himalayas. International Journal of Sustainable Development and World Ecology, 26(5), 460–470. https://doi.org/10.1080/13504509.2019.1600597
43. Dahal, Shrestha, M. L., Panthi, J., & Pradhananga, D. (2020). Modeling the future impacts of climate change on water availability in the Karnali River Basin of Nepal Himalaya. Environmental Research, 185, 109430–109430. https://doi.org/10.1016/j.envres.2020.109430
44. Kuniyal, & Sundriyal, R. C. (2013). Conservation salvage of Cordyceps sinensis collection in the Himalayan mountains is neglected. Ecosystem Services, 3, e40–e43. https://doi.org/10.1016/j.ecoser.2012.12.004
45. Negi, Maikhuri, R. ., & Rawat, L. . (2013). Ecological assessment and energy budget of fodder consumption in Govind Wildlife Sanctuary, India. International Journal of Sustainable Development and World Ecology, 20(1), 75–82. https://doi.org/10.1080/13504509.2012.747993
46. Sharma, & Vetaas, O. R. (2015). Does agroforestry conserve trees? A comparison of tree species diversity between farmland and forest in mid-hills of central Himalaya. Biodiversity and Conservation, 24(8), 2047–2061. https://doi.org/10.1007/s10531-015-0927-3
47. Rawat, Khanduri, V. P., Singh, B., Riyal, M. K., Thakur, T. K., Kumar, M., & Cabral-Pinto, M. M. (2022). Variation in carbon stock and soil properties in different Quercus leucotrichophora forests of Garhwal Himalaya. Catena (Giessen), 213, 106210–. https://doi.org/10.1016/j.catena.2022.106210
48. Tiwari. (2008). Land Use Changes in Himalaya and Their Impacts on Environment, Society and Economy： A Study of the Lake Region in Kumaon Himalaya India. Advances in Atmospheric Sciences, 25(6), 1029–1042. https://doi.org/10.1007/s00376-008-1029-x
49. Paudel, Wang, Z., Zhang, Y., Rai, M. K., & Paul, P. K. (2021). Climate Change and Its Impacts on Farmer’s Livelihood in Different Physiographic Regions of the Trans-Boundary Koshi River Basin, Central Himalayas. International Journal of Environmental Research and Public Health, 18(13), 7142–. https://doi.org/10.3390/ijerph18137142
50. Shrestha, Bajracharya, S. R., Sharma, A. R., Duo, C., & Kulkarni, A. (2017). Observed trends and changes in daily temperature and precipitation extremes over the Koshi river basin 1975–2010. International Journal of Climatology, 37(2), 1066–1083. https://doi.org/10.1002/joc.4761
51. Negi, Tiwari, D. C., Singh, L., Thakur, S., & Bhatt, I. D. (2021). Review and synthesis of climate change studies in the Himalayan region. Environment, Development and Sustainability, 24(9), 10471–10502. https://doi.org/10.1007/s10668-021-01880-5
52. Thapa, Matin, M. A., & Bajracharya, B. (2019). Capacity Building Approach and Application: Utilization of Earth Observation Data and Geospatial Information Technology in the Hindu Kush Himalaya. Frontiers in Environmental Science, 7. https://doi.org/10.3389/fenvs.2019.00165
53. Khanday, Bhat, S. U., Islam, S. T., & Sabha, I. (2021). Identifying lithogenic and anthropogenic factors responsible for spatio-seasonal patterns and quality evaluation of snow melt waters of the River Jhelum Basin in Kashmir Himalaya. Catena (Giessen), 196, 104853–. https://doi.org/10.1016/j.catena.2020.104853

Agriculture AND Trans Himalaya*

1. Baniya, Solhøy, T., & Vetaas, O. R. (2009). Temporal Changes in Species Diversity and Composition in Abandoned Fields in a Trans-Himalayan Landscape, Nepal. Plant Ecology, 201(2), 383–399. https://doi.org/10.1007/s11258-008-9473-3
2. Nüsser, Schmidt, S., & Dame, J. (2012). Irrigation and Development in the Upper Indus Basin: Characteristics and Recent Changes of a Socio-hydrological System in Central Ladakh, India. Mountain Research and Development, 32(1), 51–61. https://doi.org/10.1659/MRD-JOURNAL-D-11-00091.1
3. Mishra, Wieren, van, & Prins, H. H. . (2003). Diversity, Risk Mediation, and Change in a Trans-Himalayan Agropastoral System. Human Ecology : an Interdisciplinary Journal, 31(4), 595–609. https://doi.org/10.1023/B:HUEC.0000005515.91576.8f
4. Chauhan, Shukla, R., & Joshi, P. K. (2020). Assessing inherent vulnerability of farming communities across different biogeographical zones in Himachal Pradesh, India. Environmental Development, 33, 100506–. https://doi.org/10.1016/j.envdev.2020.100506
5. Aase, & Vetaas, O. R. (2007). Risk Management by Communal Decision in Trans-Himalayan Farming: Manang Valley in Central Nepal. Human Ecology : an Interdisciplinary Journal, 35(4), 453–460. https://doi.org/10.1007/s10745-006-9057-6
6. Aryal, Brunton, D., & Raubenheimer, D. (2014). Impact of climate change on human-wildlife-ecosystem interactions in the Trans-Himalaya region of Nepal. Theoretical and Applied Climatology, 115(3-4), 517–529. https://doi.org/10.1007/s00704-013-0902-4
7. Chettri, Sharma, E., Shakya, B., & Bajracharya, B. (2007). Developing Forested Conservation Corridors in the Kangchenjunga Landscape, Eastern Himalaya. Mountain Research and Development, 27(3), 211–214. https://doi.org/10.1659/mrd.0923
8. Nüsser, Dame, J., Parveen, S., Kraus, B., Baghel, R., & Schmidt, S. (2019). Cryosphere-Fed Irrigation Networks in the Northwestern Himalaya: Precarious Livelihoods and Adaptation Strategies Under the Impact of Climate Change. Mountain Research and Development, 39(2), R1–R11. https://doi.org/10.1659/MRD-JOURNAL-D-18-00072.1
9. Rahi, Giram, P., Chaudhari, D., diCenzo, G. C., Kiran, S., Khullar, A., Chandel, M., Gawari, S., Mohan, A., Chavan, S., & Mahajan, B. (2020). Rhizobium indicum sp. nov., isolated from root nodules of pea (Pisum sativum) cultivated in the Indian trans-Himalayas. Systematic and Applied Microbiology, 43(5), 126127–126127. https://doi.org/10.1016/j.syapm.2020.126127
10. Dame. (2018). Food Security and Translocal Livelihoods in High Mountains: Evidence from Ladakh, India. Mountain Research and Development, 38(4), 310–322. https://doi.org/10.1659/MRD-JOURNAL-D-18-00026.1
11. Gulati, Sharma, N., Vyas, P., Sood, S., Rahi, P., Pathania, V., & Prasad, R. (2010). Organic acid production and plant growth promotion as a function of phosphate solubilization by Acinetobacter rhizosphaerae strain BIHB 723 isolated from the cold deserts of the trans-Himalayas. Archives of Microbiology, 192(11), 975–983. https://doi.org/10.1007/s00203-010-0615-3
12. Paudel, Wang, Z., Zhang, Y., Rai, M. K., & Paul, P. K. (2021). Climate Change and Its Impacts on Farmer’s Livelihood in Different Physiographic Regions of the Trans-Boundary Koshi River Basin, Central Himalayas. International Journal of Environmental Research and Public Health, 18(13), 7142–. https://doi.org/10.3390/ijerph18137142
13. Bhatia, Suryawanshi, K., Redpath, S. M., & Mishra, C. (2021). Understanding people’s responses toward predators in the Indian Himalaya. Animal Conservation, 24(3), 424–431. https://doi.org/10.1111/acv.12647
14. Li, Lu, J., Wang, H., Fang, Z., Wang, X., Feng, S., Wang, Z., Yuan, T., Zhang, S., Ou, S., Yang, X., Wu, Z., Du, X., Tang, L., Liao, B., Shu, W., Jia, P., & Liang, J. (2021). A comprehensive synthesis unveils the mysteries of phosphate‐solubilizing microbes. Biological Reviews of the Cambridge Philosophical Society, 96(6), 2771–2793. https://doi.org/10.1111/brv.12779
15. Archer, Forsythe, N., Fowler, H. J., & Shah, S. M. (2010). Sustainability of water resources management in the Indus Basin under changing climatic and socio economic conditions. Hydrology and Earth System Sciences, 14(8), 1669–1680. https://doi.org/10.5194/hess-14-1669-2010
16. Lone, Jeelani, G., Deshpande, R. D., Mukherjee, A., Jasechko, S., & Lone, A. (2021). Meltwaters dominate groundwater recharge in cold arid desert of Upper Indus River Basin (UIRB), western Himalayas. The Science of the Total Environment, 786, 147514–. https://doi.org/10.1016/j.scitotenv.2021.147514
17. Xiang, Huo, S., Wang, L., Cui, L.-W., Xiao, W., Quan, R.-C., & Tai, Z. (2007). Distribution, status and conservation of the black-and-white snub-nosed monkey Rhinopithecus bieti in Tibet. Oryx, 41(4), 525–531. https://doi.org/10.1017/S0030605307012124
18. Giri, Bharti, V. K., Kalia, S., Kumar, K., Raj, T., & Chaurasia, O. P. (2019). Utility of multivariate statistical analysis to identify factors contributing river water quality in two different seasons in cold-arid high-altitude region of Leh-Ladakh, India. Applied Water Science, 9(2), 1–15. https://doi.org/10.1007/s13201-019-0902-3
19. Negi, Tiwari, D. C., Singh, L., Thakur, S., & Bhatt, I. D. (2021). Review and synthesis of climate change studies in the Himalayan region. Environment, Development and Sustainability, 24(9), 10471–10502. https://doi.org/10.1007/s10668-021-01880-5
20. Yang, Luo, W., Zhao, P., Zhang, Y., Kang, S., Giesy, J. P., & Zhang, F. (2021). Microplastics in the Koshi River, a remote alpine river crossing the Himalayas from China to Nepal. Environmental Pollution (1987), 290, 118121–118121. https://doi.org/10.1016/j.envpol.2021.118121
21. Nepal, Pradhananga, S., Shrestha, N. K., Kralisch, S., Shrestha, J. P., & Fink, M. (2021). Space-time variability in soil moisture droughts in the Himalayan region. Hydrology and Earth System Sciences, 25(4), 1761–1783. https://doi.org/10.5194/hess-25-1761-2021

Sources found using PubMed

Climate change AND Himalaya* AND Agriculture productivity

1. Paudel B, Wang Z, Zhang Y, Rai MK, Paul PK. Climate Change and Its Impacts on Farmer's Livelihood in Different Physiographic Regions of the Trans-Boundary Koshi River Basin, Central Himalayas. Int J Environ Res Public Health. 2021 Jul 3;18(13):7142. doi: 10.3390/ijerph18137142. PMID: 34281078; PMCID: PMC8296956.
2. Kumar M. Impact of climate change on crop yield and role of model for achieving food security. Environ Monit Assess. 2016 Aug;188(8):465. doi: 10.1007/s10661-016-5472-3. Epub 2016 Jul 14. PMID: 27418072.
3. Basannagari B, Kala CP. Climate change and apple farming in Indian Himalayas: a study of local perceptions and responses. PLoS One. 2013 Oct 30;8(10):e77976. doi: 10.1371/journal.pone.0077976. PMID: 24205051; PMCID: PMC3813512.
4. Mishra NB, Mainali KP. Greening and browning of the Himalaya: Spatial patterns and the role of climatic change and human drivers. Sci Total Environ. 2017 Jun 1;587-588:326-339. doi: 10.1016/j.scitotenv.2017.02.156. Epub 2017 Feb 27. PMID: 28245933.
5. Xu J, Grumbine RE, Shrestha A, Eriksson M, Yang X, Wang Y, Wilkes A. The melting Himalayas: cascading effects of climate change on water, biodiversity, and livelihoods. Conserv Biol. 2009 Jun;23(3):520-30. doi: 10.1111/j.1523-1739.2009.01237.x. PMID: 22748090.
6. Rymbai D, Sheikh FM. The insight of agricultural adaptation to climate change: a case of rice growers in Eastern Himalaya, India. Int J Biometeorol. 2018 Oct;62(10):1833-1845. doi: 10.1007/s00484-018-1586-3. Epub 2018 Aug 23. PMID: 30141151.
7. Chakraborty R, Sherpa PY. From climate adaptation to climate justice: Critical reflections on the IPCC and Himalayan climate knowledges. Clim Change. 2021;167(3-4):49. doi: 10.1007/s10584-021-03158-1. Epub 2021 Aug 23. PMID: 34456400; PMCID: PMC8381134.
8. Pandey A, Yarzábal LA. Bioprospecting cold-adapted plant growth promoting microorganisms from mountain environments. Appl Microbiol Biotechnol. 2019 Jan;103(2):643-657. doi: 10.1007/s00253-018-9515-2. Epub 2018 Nov 21. PMID: 30465306.
9. Rasul G, Hussain A, Mahapatra B, Dangol N. Food and nutrition security in the Hindu Kush Himalayan region. J Sci Food Agric. 2018 Jan;98(2):429-438. doi: 10.1002/jsfa.8530. Epub 2017 Aug 21. PMID: 28685828.
10. AbdElgawad H, Schoenaers S, Zinta G, Hassan YM, Abdel-Mawgoud M, Alkhalifah DHM, Hozzein WN, Asard H, Abuelsoud W. Soil arsenic toxicity differentially impacts C3 (barley) and C4 (maize) crops under future climate atmospheric CO_2_. J Hazard Mater. 2021 Jul 15;414:125331. doi: 10.1016/j.jhazmat.2021.125331. Epub 2021 Feb 6. PMID: 34030395.
11. Kumar R, Joshi R, Kumari M, Thakur R, Kumar D, Kumar S. Elevated CO_2_ and temperature influence key proteins and metabolites associated with photosynthesis, antioxidant and carbon metabolism in Picrorhiza kurroa. J Proteomics. 2020 May 15;219:103755. doi: 10.1016/j.jprot.2020.103755. Epub 2020 Mar 19. PMID: 32201363.
12. Ashraf A, Ahmad I. Prospects of cryosphere-fed Kuhl irrigation system nurturing high mountain agriculture under changing climate in the Upper Indus Basin. Sci Total Environ. 2021 Sep 20;788:147752. doi: 10.1016/j.scitotenv.2021.147752. Epub 2021 May 14. PMID: 34020094.
13. Pandey A, Singh G, Palni S, Chandra N, Rawat JS, Singh AP. Application of remote sensing in alpine grasslands cover mapping of western Himalaya, Uttarakhand, India. Environ Monit Assess. 2021 Mar 6;193(4):166. doi: 10.1007/s10661-021-08956-9. PMID: 33675426.
14. Anwar K, Joshi R, Dhankher OP, Singla-Pareek SL, Pareek A. Elucidating the Response of Crop Plants towards Individual, Combined and Sequentially Occurring Abiotic Stresses. Int J Mol Sci. 2021 Jun 6;22(11):6119. doi: 10.3390/ijms22116119. PMID: 34204152; PMCID: PMC8201344.
15. Mishra G, Giri K, Jangir A, Francaviglia R. Projected trends of soil organic carbon stocks in Meghalaya state of Northeast Himalayas, India. Implications for a policy perspective. Sci Total Environ. 2020 Jan 1;698:134266. doi: 10.1016/j.scitotenv.2019.134266. Epub 2019 Sep 3. PMID: 31499352.
16. Adhikari P, Jain R, Sharma A, Pandey A. Plant Growth Promotion at Low Temperature by Phosphate-Solubilizing Pseudomonas Spp. Isolated from High-Altitude Himalayan Soil. Microb Ecol. 2021 Oct;82(3):677-687. doi: 10.1007/s00248-021-01702-1. Epub 2021 Jan 29. PMID: 33512536.
17. Lal MK, Tiwari RK, Gahlaut V, Mangal V, Kumar A, Singh MP, Paul V, Kumar S, Singh B, Zinta G. Physiological and molecular insights on wheat responses to heat stress. Plant Cell Rep. 2022 Mar;41(3):501-518. doi: 10.1007/s00299-021-02784-4. Epub 2021 Sep 20. PMID: 34542670.
18. Kumar N, Jeena N, Kumar A, Khwairakpam R, Singh H. Comparative response of rice cultivars to elevated air temperature in Bhabar region of Indian Himalaya: status on yield attributes. Heliyon. 2021 Jul 5;7(7):e07474. doi: 10.1016/j.heliyon.2021.e07474. PMID: 34401552; PMCID: PMC8353292.
19. Kapoor B, Kumar A, Kumar P. Transcriptome repository of North-Western Himalayan endangered medicinal herbs: a paramount approach illuminating molecular perspective of phytoactive molecules and secondary metabolism. Mol Genet Genomics. 2021 Nov;296(6):1177-1202. doi: 10.1007/s00438-021-01821-x. Epub 2021 Sep 24. PMID: 34557965.
20. Lin YC, Zhang YL, Yu M, Fan MY, Xie F, Zhang WQ, Wu G, Cong Z, Michalski G. Formation Mechanisms and Source Apportionments of Airborne Nitrate Aerosols at a Himalayan-Tibetan Plateau Site: Insights from Nitrogen and Oxygen Isotopic Compositions. Environ Sci Technol. 2021 Sep 21;55(18):12261-12271. doi: 10.1021/acs.est.1c03957. Epub 2021 Sep 1. PMID: 34469681.
21. Rahman IU, Afzal A, Iqbal Z, Hart R, Abd Allah EF, Alqarawi AA, Alsubeie MS, Calixto ES, Ijaz F, Ali N, Kausar R, Shah M, Bussmann RW. Response of plant physiological attributes to altitudinal gradient: Plant adaptation to temperature variation in the Himalayan region. Sci Total Environ. 2020 Mar 1;706:135714. doi: 10.1016/j.scitotenv.2019.135714. Epub 2019 Nov 30. PMID: 31940726.
22. Shah S, Tiwari A, Song X, Talchabahdel R, Habiyakare T, Adhikari A. Drought index predictability for historical and future periods across the Southern plain of Nepal Himalaya. Environ Monit Assess. 2022 Aug 5;194(9):642. doi: 10.1007/s10661-022-10275-6. PMID: 35930072.
23. Goes JI, Tian H, Gomes HDR, Anderson OR, Al-Hashmi K, deRada S, Luo H, Al-Kharusi L, Al-Azri A, Martinson DG. Ecosystem state change in the Arabian Sea fuelled by the recent loss of snow over the Himalayan-Tibetan Plateau region. Sci Rep. 2020 May 4;10(1):7422. doi: 10.1038/s41598-020-64360-2. PMID: 32367063; PMCID: PMC7198515.
24. Kumar N, Jeena N, Singh H. Elevated temperature modulates rice pollen structure: a study from foothill of Himalayan agro-ecosystem in India. 3 Biotech. 2019 May;9(5):175. doi: 10.1007/s13205-019-1700-1. Epub 2019 Apr 11. PMID: 30997312; PMCID: PMC6459444.
25. Lone SA, Malik A, Padaria JC. Selection and characterization of Bacillus thuringiensis strains from northwestern Himalayas toxic against Helicoverpa armigera. Microbiologyopen. 2017 Dec;6(6):e00484. doi: 10.1002/mbo3.484. Epub 2017 Oct 18. PMID: 29047221; PMCID: PMC5727364.
26. Rahman IU, Hart RE, Ijaz F, Afzal A, Iqbal Z, Calixto ES, Abd Allah EF, Alqarawi AA, Hashem A, Al-Arjani AF, Kausar R, Haq SM. Environmental variables drive plant species composition and distribution in the moist temperate forests of Northwestern Himalaya, Pakistan. PLoS One. 2022 Feb 24;17(2):e0260687. doi: 10.1371/journal.pone.0260687. PMID: 35202409; PMCID: PMC8870539.
27. Ali MT, Mir MS, Mehraj S, Shah IA. Implications of variable environments on phenology of apple (Malus × domestica Borkh.) in Northwestern Himalayan region. Int J Biometeorol. 2022 May;66(5):945-956. doi: 10.1007/s00484-022-02250-0. Epub 2022 Feb 7. PMID: 35132442.
28. Muslim M, Romshoo SA, Rather AQ. Paddy crop yield estimation in Kashmir Himalayan rice bowl using remote sensing and simulation model. Environ Monit Assess. 2015 Jun;187(6):316. doi: 10.1007/s10661-015-4564-9. Epub 2015 May 4. PMID: 25937498.
29. Das A, Babu S, Datta M, Kumar S, Singh R, Avasthe R, Rathore SS, Yadav SK, Singh VK. Restoring soil carbon in marginal land of Indian Himalayas: Impact of crop intensification and conservation tillage. J Environ Manage. 2022 Sep 15;318:115603. doi: 10.1016/j.jenvman.2022.115603. Epub 2022 Jun 24. PMID: 35759964.
30. Debnath J, Meraj G, Das Pan N, Chand K, Debbarma S, Sahariah D, Gualtieri C, Kanga S, Singh SK, Farooq M, Sahu N, Kumar P. Integrated remote sensing and field-based approach to assess the temporal evolution and future projection of meanders: A case study on River Manu in North-Eastern India. PLoS One. 2022 Jul 20;17(7):e0271190. doi: 10.1371/journal.pone.0271190. PMID: 35857750; PMCID: PMC9299336.
31. Munawar S, Tahir MN, Baig MHA. Twenty-first century hydrologic and climatic changes over the scarcely gauged Jhelum river basin of Himalayan region using SDSM and RCPs. Environ Sci Pollut Res Int. 2022 Feb;29(8):11196-11208. doi: 10.1007/s11356-021-16437-2. Epub 2021 Sep 16. PMID: 34532792.

Climate change AND Himalaya* AND Crop yield

1. Kumar M. Impact of climate change on crop yield and role of model for achieving food security. Environ Monit Assess. 2016 Aug;188(8):465. doi: 10.1007/s10661-016-5472-3. Epub 2016 Jul 14. PMID: 27418072.
2. Muslim M, Romshoo SA, Rather AQ. Paddy crop yield estimation in Kashmir Himalayan rice bowl using remote sensing and simulation model. Environ Monit Assess. 2015 Jun;187(6):316. doi: 10.1007/s10661-015-4564-9. Epub 2015 May 4. PMID: 25937498.
3. Kumar N, Jeena N, Kumar A, Khwairakpam R, Singh H. Comparative response of rice cultivars to elevated air temperature in Bhabar region of Indian Himalaya: status on yield attributes. Heliyon. 2021 Jul 5;7(7):e07474. doi: 10.1016/j.heliyon.2021.e07474. PMID: 34401552; PMCID: PMC8353292.
4. Adhikari P, Jain R, Sharma A, Pandey A. Plant Growth Promotion at Low Temperature by Phosphate-Solubilizing Pseudomonas Spp. Isolated from High-Altitude Himalayan Soil. Microb Ecol. 2021 Oct;82(3):677-687. doi: 10.1007/s00248-021-01702-1. Epub 2021 Jan 29. PMID: 33512536.
5. Sharma RK, Shrestha DG. Climate perceptions of local communities validated through scientific signals in Sikkim Himalaya, India. Environ Monit Assess. 2016 Oct;188(10):578. doi: 10.1007/s10661-016-5582-y. Epub 2016 Sep 20. PMID: 27650439.
6. Dasgupta B, Sanyal P. Linking Land Use Land Cover change to global groundwater storage. Sci Total Environ. 2022 Sep 6:158618. doi: 10.1016/j.scitotenv.2022.158618. Epub ahead of print. PMID: 36084786.
7. Joshi S, Nath J, Singh AK, Pareek A, Joshi R. Ion transporters and their regulatory signal transduction mechanisms for salinity tolerance in plants. Physiol Plant. 2022 May;174(3):e13702. doi: 10.1111/ppl.13702. PMID: 35524987.
8. Malhotra N, Sharma P, Sood H, Chandora R, Arya M, Rana JC, Singh M. Agro-Morphological Characterization and Nutritional Profiling of Traditional Himalayan Crop Landraces for Their Promotion Toward Mainstream Agriculture. Front Plant Sci. 2022 Jun 22;13:898220. doi: 10.3389/fpls.2022.898220. PMID: 35812955; PMCID: PMC9258745.
9. Dutta M, Raturi V, Gahlaut V, Kumar A, Sharma P, Verma V, Gupta VK, Sood S, Zinta G. The interplay of DNA methyltransferases and demethylases with tuberization genes in potato (*Solanum tuberosum* L.) genotypes under high temperature. Front Plant Sci. 2022 Aug 16;13:933740. doi: 10.3389/fpls.2022.933740. PMID: 36051291; PMCID: PMC9425917.

Climate change AND Himalaya* AND Crop suitability

1. Paudel B, Wang Z, Zhang Y, Rai MK, Paul PK. Climate Change and Its Impacts on Farmer's Livelihood in Different Physiographic Regions of the Trans-Boundary Koshi River Basin, Central Himalayas. Int J Environ Res Public Health. 2021 Jul 3;18(13):7142. doi: 10.3390/ijerph18137142. PMID: 34281078; PMCID: PMC8296956.
2. Ashraf A, Ahmad I. Prospects of cryosphere-fed Kuhl irrigation system nurturing high mountain agriculture under changing climate in the Upper Indus Basin. Sci Total Environ. 2021 Sep 20;788:147752. doi: 10.1016/j.scitotenv.2021.147752. Epub 2021 May 14. PMID: 34020094.

Climate change AND Himalaya* AND Ecosystem service*

1. Gurung LJ, Miller KK, Venn S, Bryan BA. Climate change adaptation for managing non-timber forest products in the Nepalese Himalaya. Sci Total Environ. 2021 Nov 20;796:148853. doi: 10.1016/j.scitotenv.2021.148853. Epub 2021 Jul 3. PMID: 34265618.
2. Aukema JE, Pricope NG, Husak GJ, Lopez-Carr D. Biodiversity Areas under Threat: Overlap of Climate Change and Population Pressures on the World's Biodiversity Priorities. PLoS One. 2017 Jan 26;12(1):e0170615. doi: 10.1371/journal.pone.0170615. PMID: 28125659; PMCID: PMC5268772.
3. Dahal P, Shrestha ML, Panthi J, Pradhananga D. Modeling the future impacts of climate change on water availability in the Karnali River Basin of Nepal Himalaya. Environ Res. 2020 Jun;185:109430. doi: 10.1016/j.envres.2020.109430. Epub 2020 Mar 28. PMID: 32247907.
4. Romshoo SA, Murtaza KO, Shah W, Ramzan T, Ameen U, Bhat MH. Anthropogenic climate change drives melting of glaciers in the Himalaya. Environ Sci Pollut Res Int. 2022 Jul;29(35):52732-52751. doi: 10.1007/s11356-022-19524-0. Epub 2022 Mar 10. PMID: 35274205.
5. Wani IA, Khan S, Verma S, Al-Misned FA, Shafik HM, El-Serehy HA. Predicting habitat suitability and niche dynamics of Dactylorhiza hatagirea and Rheum webbianum in the Himalaya under projected climate change. Sci Rep. 2022 Aug 1;12(1):13205. doi: 10.1038/s41598-022-16837-5. PMID: 35915126; PMCID: PMC9343649.
6. Reang D, Hazarika A, Sileshi GW, Pandey R, Das AK, Nath AJ. Assessing tree diversity and carbon storage during land use transitioning from shifting cultivation to indigenous agroforestry systems: Implications for REDD+ initiatives. J Environ Manage. 2021 Nov 15;298:113470. doi: 10.1016/j.jenvman.2021.113470. Epub 2021 Aug 6. PMID: 34365182.
7. Lamsal P, Kumar L, Aryal A, Atreya K. Invasive alien plant species dynamics in the Himalayan region under climate change. Ambio. 2018 Oct;47(6):697-710. doi: 10.1007/s13280-018-1017-z. Epub 2018 Jan 25. PMID: 29372502; PMCID: PMC6131134.
8. Pandey A, Singh G, Palni S, Chandra N, Rawat JS, Singh AP. Application of remote sensing in alpine grasslands cover mapping of western Himalaya, Uttarakhand, India. Environ Monit Assess. 2021 Mar 6;193(4):166. doi: 10.1007/s10661-021-08956-9. PMID: 33675426.
9. Reddy CS, Satish KV, Jha CS, Diwakar PG, Murthy YV, Dadhwal VK. Development of deforestation and land cover database for Bhutan (1930-2014). Environ Monit Assess. 2016 Dec;188(12):658. doi: 10.1007/s10661-016-5676-6. Epub 2016 Nov 10. PMID: 27832432.
10. Xu J, Sharma R, Fang J, Xu Y. Critical linkages between land-use transition and human health in the Himalayan region. Environ Int. 2008 Feb;34(2):239-47. doi: 10.1016/j.envint.2007.08.004. Epub 2007 Sep 14. PMID: 17868868.
11. Dorji S, Vernes K, Rajaratnam R. Habitat correlates of the red panda in the temperate forests of Bhutan. PLoS One. 2011;6(10):e26483. doi: 10.1371/journal.pone.0026483. Epub 2011 Oct 19. PMID: 22039497; PMCID: PMC3198399.
12. Zhang ML, Xiang XG, Xue JJ, Sanderson SC, Fritsch PW. Himalayan uplift shaped biomes in Miocene temperate Asia: evidence from leguminous Caragana. Sci Rep. 2016 Nov 9;6:36528. doi: 10.1038/srep36528. PMID: 27827446; PMCID: PMC5101512.

Agriculture AND Himalaya* AND Ecosystem service*

1. Aryal KP, Poudel S, Chaudhary RP, Chettri N, Chaudhary P, Ning W, Kotru R. Diversity and use of wild and non-cultivated edible plants in the Western Himalaya. J Ethnobiol Ethnomed. 2018 Jan 29;14(1):10. doi: 10.1186/s13002-018-0211-1. PMID: 29378614; PMCID: PMC5789610.
2. Reang D, Hazarika A, Sileshi GW, Pandey R, Das AK, Nath AJ. Assessing tree diversity and carbon storage during land use transitioning from shifting cultivation to indigenous agroforestry systems: Implications for REDD+ initiatives. J Environ Manage. 2021 Nov 15;298:113470. doi: 10.1016/j.jenvman.2021.113470. Epub 2021 Aug 6. PMID: 34365182.
3. Dorji S, Rajaratnam R, Falconi L, Williams SE, Sinha P, Vernes K. Identifying conservation priorities for threatened Eastern Himalayan mammals. Conserv Biol. 2018 Oct;32(5):1162-1173. doi: 10.1111/cobi.13115. Epub 2018 Aug 25. Erratum in: Conserv Biol. 2019 Feb;33(1):225. PMID: 30055016.
4. Grace MK, Akçakaya HR, Bennett EL, Brooks TM, Heath A, Hedges S, Hilton-Taylor C, Hoffmann M, Hochkirch A, Jenkins R, Keith DA, Long B, Mallon DP, Meijaard E, Milner-Gulland EJ, Rodriguez JP, Stephenson PJ, Stuart SN, Young RP, Acebes P, Alfaro-Shigueto J, Alvarez-Clare S, Andriantsimanarilafy RR, Arbetman M, Azat C, Bacchetta G, Badola R, Barcelos LMD, Barreiros JP, Basak S, Berger DJ, Bhattacharyya S, Bino G, Borges PAV, Boughton RK, Brockmann HJ, Buckley HL, Burfield IJ, Burton J, Camacho-Badani T, Cano-Alonso LS, Carmichael RH, Carrero C, Carroll JP, Catsadorakis G, Chapple DG, Chapron G, Chowdhury GW, Claassens L, Cogoni D, Constantine R, Craig CA, Cunningham AA, Dahal N, Daltry JC, Das GC, Dasgupta N, Davey A, Davies K, Develey P, Elangovan V, Fairclough D, Febbraro MD, Fenu G, Fernandes FM, Fernandez EP, Finucci B, Földesi R, Foley CM, Ford M, Forstner MRJ, García N, Garcia-Sandoval R, Gardner PC, Garibay-Orijel R, Gatan-Balbas M, Gauto I, Ghazi MGU, Godfrey SS, Gollock M, González BA, Grant TD, Gray T, Gregory AJ, van Grunsven RHA, Gryzenhout M, Guernsey NC, Gupta G, Hagen C, Hagen CA, Hall MB, Hallerman E, Hare K, Hart T, Hartdegen R, Harvey-Brown Y, Hatfield R, Hawke T, Hermes C, Hitchmough R, Hoffmann PM, Howarth C, Hudson MA, Hussain SA, Huveneers C, Jacques H, Jorgensen D, Katdare S, Katsis LKD, Kaul R, Kaunda-Arara B, Keith-Diagne L, Kraus DT, de Lima TM, Lindeman K, Linsky J, Louis E Jr, Loy A, Lughadha EN, Mangel JC, Marinari PE, Martin GM, Martinelli G, McGowan PJK, McInnes A, Teles Barbosa Mendes E, Millard MJ, Mirande C, Money D, Monks JM, Morales CL, Mumu NN, Negrao R, Nguyen AH, Niloy MNH, Norbury GL, Nordmeyer C, Norris D, O'Brien M, Oda GA, Orsenigo S, Outerbridge ME, Pasachnik S, Pérez-Jiménez JC, Pike C, Pilkington F, Plumb G, Portela RCQ, Prohaska A, Quintana MG, Rakotondrasoa EF, Ranglack DH, Rankou H, Rawat AP, Reardon JT, Rheingantz ML, Richter SC, Rivers MC, Rogers LR, da Rosa P, Rose P, Royer E, Ryan C, de Mitcheson YJS, Salmon L, Salvador CH, Samways MJ, Sanjuan T, Souza Dos Santos A, Sasaki H, Schutz E, Scott HA, Scott RM, Serena F, Sharma SP, Shuey JA, Silva CJP, Simaika JP, Smith DR, Spaet JLY, Sultana S, Talukdar BK, Tatayah V, Thomas P, Tringali A, Trinh-Dinh H, Tuboi C, Usmani AA, Vasco-Palacios AM, Vié JC, Virens J, Walker A, Wallace B, Waller LJ, Wang H, Wearn OR, van Weerd M, Weigmann S, Willcox D, Woinarski J, Yong JWH, Young S. Testing a global standard for quantifying species recovery and assessing conservation impact. Conserv Biol. 2021 Dec;35(6):1833-1849. doi: 10.1111/cobi.13756. Epub 2021 Jul 21. PMID: 34289517.
5. Pandey A, Singh G, Palni S, Chandra N, Rawat JS, Singh AP. Application of remote sensing in alpine grasslands cover mapping of western Himalaya, Uttarakhand, India. Environ Monit Assess. 2021 Mar 6;193(4):166. doi: 10.1007/s10661-021-08956-9. PMID: 33675426.
6. Reddy CS, Satish KV, Jha CS, Diwakar PG, Murthy YV, Dadhwal VK. Development of deforestation and land cover database for Bhutan (1930-2014). Environ Monit Assess. 2016 Dec;188(12):658. doi: 10.1007/s10661-016-5676-6. Epub 2016 Nov 10. PMID: 27832432.
7. Chakraborty P, Shappell NW, Mukhopadhyay M, Onanong S, Rex KR, Snow D. Surveillance of plasticizers, bisphenol A, steroids and caffeine in surface water of River Ganga and Sundarban wetland along the Bay of Bengal: occurrence, sources, estrogenicity screening and ecotoxicological risk assessment. Water Res. 2021 Feb 15;190:116668. doi: 10.1016/j.watres.2020.116668. Epub 2020 Nov 23. PMID: 33285458.
8. Malik M, Rai SC. Drivers of land use/cover change and its impact on Pong Dam wetland. Environ Monit Assess. 2019 Mar 4;191(4):203. doi: 10.1007/s10661-019-7347-x. PMID: 30834470.
9. Lamsal P, Kumar L, Aryal A, Atreya K. Invasive alien plant species dynamics in the Himalayan region under climate change. Ambio. 2018 Oct;47(6):697-710. doi: 10.1007/s13280-018-1017-z. Epub 2018 Jan 25. PMID: 29372502; PMCID: PMC6131134.
10. Aukema JE, Pricope NG, Husak GJ, Lopez-Carr D. Biodiversity Areas under Threat: Overlap of Climate Change and Population Pressures on the World's Biodiversity Priorities. PLoS One. 2017 Jan 26;12(1):e0170615. doi: 10.1371/journal.pone.0170615. PMID: 28125659; PMCID: PMC5268772.
11. Pollegioni P, Lungo SD, Müller R, Woeste KE, Chiocchini F, Clark J, Hemery GE, Mapelli S, Villani F, Malvolti ME, Mattioni C. Biocultural diversity of common walnut (*Juglans regia* L.) and sweet chestnut (*Castanea sativa* Mill.) across Eurasia. Ecol Evol. 2020 Sep 24;10(20):11192-11216. doi: 10.1002/ece3.6761. PMID: 33144959; PMCID: PMC7593191.
12. Zhang ML, Xiang XG, Xue JJ, Sanderson SC, Fritsch PW. Himalayan uplift shaped biomes in Miocene temperate Asia: evidence from leguminous Caragana. Sci Rep. 2016 Nov 9;6:36528. doi: 10.1038/srep36528. PMID: 27827446; PMCID: PMC5101512.
13. Dorji S, Vernes K, Rajaratnam R. Habitat correlates of the red panda in the temperate forests of Bhutan. PLoS One. 2011;6(10):e26483. doi: 10.1371/journal.pone.0026483. Epub 2011 Oct 19. PMID: 22039497; PMCID: PMC3198399.
14. Dahal P, Shrestha ML, Panthi J, Pradhananga D. Modeling the future impacts of climate change on water availability in the Karnali River Basin of Nepal Himalaya. Environ Res. 2020 Jun;185:109430. doi: 10.1016/j.envres.2020.109430. Epub 2020 Mar 28. PMID: 32247907.
15. Xu J, Sharma R, Fang J, Xu Y. Critical linkages between land-use transition and human health in the Himalayan region. Environ Int. 2008 Feb;34(2):239-47. doi: 10.1016/j.envint.2007.08.004. Epub 2007 Sep 14. PMID: 17868868.
16. Zahid M, Abbasi MK, Hameed S, Rahim N. Isolation and identification of indigenous plant growth promoting rhizobacteria from Himalayan region of Kashmir and their effect on improving growth and nutrient contents of maize (Zea mays L.). Front Microbiol. 2015 Mar 17;6:207. doi: 10.3389/fmicb.2015.00207. PMID: 25852667; PMCID: PMC4362393.
17. Garibaldi LA, Carvalheiro LG, Vaissière BE, Gemmill-Herren B, Hipólito J, Freitas BM, Ngo HT, Azzu N, Sáez A, Åström J, An J, Blochtein B, Buchori D, Chamorro García FJ, Oliveira da Silva F, Devkota K, Ribeiro Mde F, Freitas L, Gaglianone MC, Goss M, Irshad M, Kasina M, Pacheco Filho AJ, Kiill LH, Kwapong P, Parra GN, Pires C, Pires V, Rawal RS, Rizali A, Saraiva AM, Veldtman R, Viana BF, Witter S, Zhang H. Mutually beneficial pollinator diversity and crop yield outcomes in small and large farms. Science. 2016 Jan 22;351(6271):388-91. doi: 10.1126/science.aac7287. PMID: 26798016.
18. Neemisha, Kumar A, Sharma P, Kaur A, Sharma S, Jain R. Harnessing rhizobacteria to fulfil inter-linked nutrient dependency on soil and alleviate stresses in plants. J Appl Microbiol. 2022 Jun 3. doi: 10.1111/jam.15649. Epub ahead of print. PMID: 35656999.
19. Tambe S, Rawat GS. Ecology, economics, and equity of the pastoral systems in the Khangchendzonga National Park, Sikkim Himalaya, India. Ambio. 2009 Mar;38(2):95-100. doi: 10.1579/0044-7447-38.2.95. PMID: 19431939.

Agriculture AND Trans Himalaya*

1. Majeed U, Rashid I, Sattar A, Allen S, Stoffel M, Nüsser M, Schmidt S. Recession of Gya Glacier and the 2014 glacial lake outburst flood in the Trans-Himalayan region of Ladakh, India. Sci Total Environ. 2021 Feb 20;756:144008. doi: 10.1016/j.scitotenv.2020.144008. Epub 2020 Nov 27. PMID: 33293089.
2. Paudel B, Wang Z, Zhang Y, Rai MK, Paul PK. Climate Change and Its Impacts on Farmer's Livelihood in Different Physiographic Regions of the Trans-Boundary Koshi River Basin, Central Himalayas. Int J Environ Res Public Health. 2021 Jul 3;18(13):7142. doi: 10.3390/ijerph18137142. PMID: 34281078; PMCID: PMC8296956.
3. Namgail T, Fox JL, Bhatnagar YV. Carnivore-caused livestock mortality in Trans-Himalaya. Environ Manage. 2007 Apr;39(4):490-6. doi: 10.1007/s00267-005-0178-2. PMID: 17318699.
4. Kumar P, Kumar P, Sharma M, Shukla AK, Butail NP. Spatial variability of soil nutrients in apple orchards and agricultural areas in Kinnaur region of cold desert, Trans-Himalaya, India. Environ Monit Assess. 2022 Mar 23;194(4):290. doi: 10.1007/s10661-022-09936-3. PMID: 35320425.
5. Yang L, Luo W, Zhao P, Zhang Y, Kang S, Giesy JP, Zhang F. Microplastics in the Koshi River, a remote alpine river crossing the Himalayas from China to Nepal. Environ Pollut. 2021 Dec 1;290:118121. doi: 10.1016/j.envpol.2021.118121. Epub 2021 Sep 7. PMID: 34523512.
6. Haq SM, Yaqoob U, Calixto ES, Rahman IU, Hashem A, Abd Allah EF, Alakeel MA, Alqarawi AA, Abdalla M, Hassan M, Bussmann RW, Abbasi AM, Ur Rahman S, Ijaz F. Plant Resources Utilization among Different Ethnic Groups of Ladakh in Trans-Himalayan Region. Biology (Basel). 2021 Aug 26;10(9):827. doi: 10.3390/biology10090827. PMID: 34571704; PMCID: PMC8468708.
7. Bhatnagar YV, Wangchuk R, Prins HH, Van Wieren SE, Mishra C. Perceived conflicts between pastoralism and conservation of the kiang Equus kiang in the Ladakh Trans-Himalaya, India. Environ Manage. 2006 Dec;38(6):934-41. doi: 10.1007/s00267-005-0356-2. Epub 2006 Sep 2. PMID: 16955231; PMCID: PMC1705511.
8. Tyagi R, Tiwari A, Gupta AK, Gupta S. Transcriptome wide identification and characterization of Starch Synthase enzyme in finger millet. Bioinformation. 2018 Jul 31;14(7):393-397. doi: 10.6026/97320630014393. PMID: 30262977; PMCID: PMC6143355.
9. Pollegioni P, Lungo SD, Müller R, Woeste KE, Chiocchini F, Clark J, Hemery GE, Mapelli S, Villani F, Malvolti ME, Mattioni C. Biocultural diversity of common walnut (*Juglans regia* L.) and sweet chestnut (*Castanea sativa* Mill.) across Eurasia. Ecol Evol. 2020 Sep 24;10(20):11192-11216. doi: 10.1002/ece3.6761. PMID: 33144959; PMCID: PMC7593191.
10. Wiley AS. Neonatal and maternal anthropometric characteristics in a high altitude population of the western Himalaya. Am J Hum Biol. 1994;6(4):499-510. doi: 10.1002/ajhb.1310060411. PMID: 28548251.
11. Lamsal P, Kumar L, Aryal A, Atreya K. Invasive alien plant species dynamics in the Himalayan region under climate change. Ambio. 2018 Oct;47(6):697-710. doi: 10.1007/s13280-018-1017-z. Epub 2018 Jan 25. PMID: 29372502; PMCID: PMC6131134.
12. Baniya B, Tang Q, Xu X, Haile GG, Chhipi-Shrestha G. Spatial and Temporal Variation of Drought Based on Satellite Derived Vegetation Condition Index in Nepal from 1982⁻2015. Sensors (Basel). 2019 Jan 21;19(2):430. doi: 10.3390/s19020430. PMID: 30669648; PMCID: PMC6359269.
13. Chatli AS, Beri V, Sidhu BS. Isolation and characterisation of phosphate solubilising microorganisms from the cold desert habitat of Salix alba Linn. in trans Himalayan region of Himachal Pradesh. Indian J Microbiol. 2008 Jun;48(2):267-73. doi: 10.1007/s12088-008-0037-y. Epub 2008 Jul 27. PMID: 23100719; PMCID: PMC3450174.
14. Rahi P, Giram P, Chaudhari D, diCenzo GC, Kiran S, Khullar A, Chandel M, Gawari S, Mohan A, Chavan S, Mahajan B. Rhizobium indicum sp. nov., isolated from root nodules of pea (Pisum sativum) cultivated in the Indian trans-Himalayas. Syst Appl Microbiol. 2020 Sep;43(5):126127. doi: 10.1016/j.syapm.2020.126127. Epub 2020 Jul 30. PMID: 32847793.
15. Gulati A, Sharma N, Vyas P, Sood S, Rahi P, Pathania V, Prasad R. Organic acid production and plant growth promotion as a function of phosphate solubilization by Acinetobacter rhizosphaerae strain BIHB 723 isolated from the cold deserts of the trans-Himalayas. Arch Microbiol. 2010 Nov;192(11):975-83. doi: 10.1007/s00203-010-0615-3. Epub 2010 Sep 7. PMID: 20821196.
16. Moudi M, Galoie M, Yuan H, Motamedi A, Huang P, Shafi M. Dynamic multi-objective programming model for improving consumer satisfaction within water supply system under uncertain environment. J Environ Manage. 2021 Sep 1;293:112897. doi: 10.1016/j.jenvman.2021.112897. Epub 2021 Jun 1. PMID: 34082342.
17. Mehmood MA, Qadri H, Bhat RA, Rashid A, Ganie SA, Dar GH, Shafiq-Ur-Rehman. Heavy metal contamination in two commercial fish species of a trans-Himalayan freshwater ecosystem. Environ Monit Assess. 2019 Jan 26;191(2):104. doi: 10.1007/s10661-019-7245-2. PMID: 30685798.
18. Verma P, Sharma A, Sodhi M, Thakur K, Bharti VK, Kumar P, Giri A, Kalia S, Swami SK, Mukesh M. Overexpression of genes associated with hypoxia in cattle adapted to Trans Himalayan region of Ladakh. Cell Biol Int. 2018 Sep;42(9):1141-1148. doi: 10.1002/cbin.10981. Epub 2018 Jul 11. PMID: 29719086.
19. Chetri M, Odden M, Devineau O, McCarthy T, Wegge P. Multiple factors influence local perceptions of snow leopards and Himalayan wolves in the central Himalayas, Nepal. PeerJ. 2020 Oct 15;8:e10108. doi: 10.7717/peerj.10108. PMID: 33088621; PMCID: PMC7568854.
20. Baylis M, Barker CM, Caminade C, Joshi BR, Pant GR, Rayamajhi A, Reisen WK, Impoinvil DE. Emergence or improved detection of Japanese encephalitis virus in the Himalayan highlands? Trans R Soc Trop Med Hyg. 2016 Apr;110(4):209-11. doi: 10.1093/trstmh/trw012. Epub 2016 Mar 7. PMID: 26956778; PMCID: PMC4830403.
21. Farooqi MK, Ali M, Amir M, Usmani MK. First record of grey bush-cricket Platycleis albopunctata (Goeze, 1778) (Tettigoniidae: Tettigoniini) on the agricultural crops from Trans Himalaya, India. Zootaxa. 2022 Mar 28;5120(3):435-442. doi: 10.11646/zootaxa.5120.3.9. PMID: 35391157.
22. Suryawanshi KR, Bhatia S, Bhatnagar YV, Redpath S, Mishra C. Multiscale factors affecting human attitudes toward snow leopards and wolves. Conserv Biol. 2014 Dec;28(6):1657-66. doi: 10.1111/cobi.12320. Epub 2014 Jul 15. PMID: 25039397.
23. Wang Z, Da W, Negi CS, Ghimire PL, Wangdi K, Yadav PK, Pubu Z, Lama L, Yarpel K, Maunsell SC, Liu Y, Kunte K, Bawa KS, Yang D, Pierce NE. Profiling, monitoring and conserving caterpillar fungus in the Himalayan region using anchored hybrid enrichment markers. Proc Biol Sci. 2022 Apr 27;289(1973):20212650. doi: 10.1098/rspb.2021.2650. Epub 2022 Apr 27. PMID: 35473372; PMCID: PMC9043734.
24. Muslim M, Romshoo SA, Rather AQ. Paddy crop yield estimation in Kashmir Himalayan rice bowl using remote sensing and simulation model. Environ Monit Assess. 2015 Jun;187(6):316. doi: 10.1007/s10661-015-4564-9. Epub 2015 May 4. PMID: 25937498.
25. Giri A, Bharti VK, Kalia S, Acharya S, Kumar B, Chaurasia OP. Health Risk Assessment of Heavy Metals Due to Wheat, Cabbage, and Spinach Consumption at Cold-Arid High Altitude Region. Biol Trace Elem Res. 2022 Sep;200(9):4186-4198. doi: 10.1007/s12011-021-03006-4. Epub 2021 Nov 8. PMID: 34750742.
26. Luo W, Gao J, Bi X, Xu L, Guo J, Zhang Q, Romesh KY, Giesy JP, Kang S. Identification of sources of polycyclic aromatic hydrocarbons based on concentrations in soils from two sides of the Himalayas between China and Nepal. Environ Pollut. 2016 May;212:424-432. doi: 10.1016/j.envpol.2015.11.018. Epub 2016 Feb 21. PMID: 26900777.
